# Postoperative tight glycemic control significantly reduces postoperative infection rates in patients undergoing surgery: a meta-analysis

**DOI:** 10.1186/s12902-018-0268-9

**Published:** 2018-06-22

**Authors:** Yuan-yuan Wang, Shuang-fei Hu, Hui-min Ying, Long Chen, Hui-li Li, Fang Tian, Zhen-feng Zhou

**Affiliations:** 1 Department of Endocrinology, Xixi Hospital of Hangzhou, Hangzhou, Hangzhou, 315000 Zhejiang Province China; 2 Department of Anesthesiology, Zhejiang Provincial People’s Hospital (People’s Hospital of Hangzhou Medicine College), Hangzhou, 315000 China

**Keywords:** Tight glycemic control, Postoperative, Infection

## Abstract

**Background:**

The benefit results of postoperative tight glycemic control (TGC) were controversial and there was a lack of well-powered studies that support current guideline recommendations.

**Methods:**

The EMBASE, MEDLINE, and the Cochrane Library databases were searched utilizing the key words “Blood Glucose”, “insulin” and “Postoperative Period” to retrieve all randomized controlled trials evaluating the benefits of postoperative TGC as compared to conventional glycemic control (CGC) in patients undergoing surgery.

**Results:**

Fifteen studies involving 5053 patients were identified. As compared to CGC group, there were lower risks of total postoperative infection (9.4% vs. 15.8%; RR 0.586, 95% CI 0.504 to 0.680, *p* <  0.001) and wound infection (4.6% vs. 7.2%; RR 0.620, 95% CI 0.422 to 0.910, *p* = 0.015) in TGC group. TGC also showed a lower risk of postoperative short-term mortality (3.8% vs. 5.4%; RR 0.692, 95% CI 0.527 to 0.909, *p* = 0.008), but sensitivity analyses showed that the result was mainly influenced by one study. The patients in the TGC group experienced a significant higher rate of postoperative hypoglycemia (22.3% vs. 11.0%; RR 3.145, 95% CI 1.928 to 5.131, *p* <  0.001) and severe hypoglycemia (2.8% vs. 0.7%; RR 3.821, 95% CI 1.796 to 8.127, *p* <  0.001) as compared to CGC group. TGC showed less length of ICU stay (SMD, − 0.428 days; 95% CI, − 0.833 to − 0.022 days; *p* = 0.039). However, TGC showed a neutral effect on neurological dysfunction (1.1% vs. 2.4%; RR 0.499, 95% CI 0.219 to 1.137, *p* = 0.098), acute renal failure (3.3% vs. 5.4%, RR 0.610, 95% CI 0.359 to 1.038, *p* = 0.068), duration of mechanical ventilation (*p* = 0.201) and length of hospitalization (*p* = 0.082).

**Conclusions:**

TGC immediately after surgery significantly reduces total postoperative infection rates and short-term mortality. However, it might limit conclusion regarding the efficacy of TGC for short-term mortality in sensitivity analyses. The patients in the TGC group experienced a significant higher rate of postoperative hypoglycemia. This study may suggest that TGC should be administrated under close glucose monitoring in patients undergoing surgery, especially in those with high postoperative infection risk.

**Electronic supplementary material:**

The online version of this article (10.1186/s12902-018-0268-9) contains supplementary material, which is available to authorized users.

## Background

Tight glycemic control (TGC) was found to decrease the mortality and morbidity in critically ill patients [1] and it has therefore been recommended as the standard treatment for the duration of the perioperative intensive care unit (ICU) throughout the world. However, subsequent trials have failed to confirm the benefits of this recommendation [2, 3].

Perioperative hyperglycemia is reported in approximately 20–40% of patients after general surgery [4] and almost 80% of patients undergoing cardiac surgery [5]. Several studies in cardiac surgery and general surgery have shown a clear association between perioperative hyperglycemia and adverse clinical outcomes including delayed wound healing, surgical site infections, and prolonged hospital stay [4, 6]. However, the optimal glucose target during the post-operative period is widely controversial. No significant difference was found between TGC and conventional glycemic control (CGC) when evaluating the variety of complications [5, 7, 8]. However, another study. [9] including cardiac surgery patients reported a reduction of postoperative complications in TGC group.

Given the conflicting results and the lack of well-powered studies that support current guideline recommendations, the present study employed meta-analysis to evaluate the current evidence and analyze the association between the strategies of postoperative glycemic control and outcomes in patients undergoing elective surgery.

## Methods

This meta-analysis was performed according to meta-analyses (PRISMA) format guidelines [10].

### Search strategy

EMBASE, MEDLINE, and the Cochrane Library were searched electronically by two investigators (Long CHEN and Fang TIAN) for relevant studies, and the following key words were used: “Blood Glucose” “insulin” and “Postoperative Period”. The searches were last updated in 16th April 2018. Search strategies are available in Additional file 1. Two investigators (Long CHEN and Fang TIAN) independently screened the titles and abstracts to exclude irrelevant articles. Then, they reviewed the full-text articles to ensure all relevant articles had been included. A third author resolved any controversies.

### Study selection and data extraction

We only included randomized controlled trials (RCTs) with surgery patients who had received postoperative TGC. Specific eligibility criteria were as follows: (a) published in English; (b) treatment with postoperative TGC; and (c) the study documented any endpoints including infection or mortality. Two authors (Long CHEN and Fang TIAN) evaluated all records according to the above eligibility criteria (Table 1). We abstracted the year of publication, sample size, type of surgery, population type (adult or infant), patient age, gender, history of diabetes, baseline BG (blood glucose level), time of TGC intervention (only during the post-operative or intra-operative plus post-operative), target BG and trigger BG for intervention, any insulin infusion protocol, and reported clinical outcomes.

**Table 1:** Main characteristics of the included trials

First author	Type of surgery		Patient type	Sample	Time of intervention		Insulin infusion protocol	Mean age (mean ± SD, year)	Male (%)		BMI(mean ± SD)		
				TGC/CGC				TGC	CGC	TGC	CGC	TGC	CGC
Van Den et al. (2001)	various surgeries		adult	1548(765/783)	postoperative		Yes	63.4 ± 13.6	62.2 ± 13.9	71	71	26.2 ± 4.4	25.8 ± 4.7
Salah M et al. (2013)	cardiac surgery		adult	100(50/50)	Intra + post operative		Yes	46.0 ± 9.0	49.0 ± 8.0	60	70	28.0 ± 0.8	26.0 ± 2.0
Konstantinos et al. (2013)	cardiac surgery		adult	212(105/107)	postoperative		Yes	64.9 ± 11.5	66.9 ± 11.1	66	68	27.8 ± 4.4	28.0 ± 3.9
Amisha et al. (2017)	liver transplantation		adult	164(82/82)	postoperative		Yes	58.1 ± 8.0	56.9 ± 7.6	65	65	30.3 ± 6.0	30.0 ± 6.6
Rehong Zheng et al. (2010)	cardiac surgery		adult	100(50/50)	Intra + post operative		Yes	43.3 ± 11.7	44.0 ± 11.5	48	46		
Raquel Pei Chen Chan et al. (2009)	cardiac surgery		adult	108(54/55)	Intra + post operative		Yes	57.0 ± 12.0	58 ± 12	43	56	24.0 ± 3.4	26.0 ± 4.9
Shou-gen Cao et al. (2011)	elective radical gastrectomy with D2 lymphadenectomy		adult	248(125/123)	postoperative		Yes	58.5 ± 8.1	59.9 ± 7.6	67	64	20.8 ± 2.1	21.2 ± 2.1
Shou-gen Cao et al. (2011)	elective radical gastrectomy with D2 lymphadenectomy		adult	179(92/87)	postoperative		Yes	58.2 ± 6.3	59.4 ± 7.3	30	34	21.1 ± 2.0	22.2 ± 2.6
Takehiro Okabayashi et al. (2014)	hepatectomy or pancreatectomy		adult	447(222/225)	Intra + post operative		No	66.7 ± 10.1	66.4 ± 10.4	64	67	23.3 ± 3.6	23.1 ± 3.4
Ehab A. Wahby et al. (2016)	cardiac surgery		adult	135(67/68)	Intra + post operative		No	54.99 ± 6.49	56.40 ± 7.79	73	68		
Federico Bilotta et al. (2009)	Neurosurgical		adult	483(241/242)	postoperative		Yes	57.3 ± 11.9	56.9 ± 12.7	63	52		
Shalin P. Desai et al. (2012)	cardiac surgery		adult	189(91/98)	postoperative		Yes	62.5 ± 10.2	62.8 ± 9.5	89	80		
Federico Bilotta et al. (2007)	Intracranial Aneurysm Clipping		adult	78(40/38)	Intra + post operative		Yes	53.0 ± 16.0	52.0 ± 15.0	30	32		
Michael SD Agus et al. (2012)	cardiac surgery		birth to 36 months	980(490/490)	postoperative		Yes	4.3(1.8–9.7) months	4.9(2.3–10.8) months	51	56		
Harold L et al. (2011)	cardiac surgery		adult	82(40/42)	Intra + post operative		Yes	63 ± 9	65 ± 9	65	76		
First author	History of diabetes (%)		Baseline blood glucose (mean ± SD, mg/dL)		Target blood glucose(mean ± SD, mg/dL)		Trigger blood glucose(mean ± SD, mg/dL)		Postoperatively maintain blood glucose(mean ± SD, mg/dl)		Glucocorti-coids used	postoperative follow-up	Jadad Score
	TGC	CGC	TGC	CGC	TGC	CGC	TGC	CGC	TGC	CGC			
Van Den et al. (2001)	13	13			80–110	180–200	110	215	103 ± 19	153 ± 33	No	in-hospital	6
Salah M et al. (2013)	100	100			80–110	110–180	110	180			No	6 months	2
Konstantinos et al. (2013)	26	31			120–160	161–200	160	200	153.9 ± 13.9	173.9 ± 17.4	No	30 days or in-hospital	1
Amisha et al. (2017)	28	32					140	180	146.4 ± 16.4	178.8 ± 24.0	Yes	1 year	4
Rehong Zheng et al. (2010)	0	0	78.4 ± 3.8	77.5 ± 2.6	70–110		110				No		2
Raquel Pei Chen Chan et al. (2009)			113.4 ± 42.5	138 ± 75	80–130	160–200	130	200	126.7 ± 10.8	168.2 ± 28.3	No	30 days	3
Shou-gen Cao et al. (2011)	0	0	99.0 ± 14.4	95 ± 13	80–110	< 200	110	200	94.5 ± 15.3	184.0 ± 16.7	No	28 days	2
Shou-gen Cao et al. (2011)	100	100	122.4 ± 10.8	126 ± 13	80–110	180–215	110	215	99 ± 14.4	178.2 ± 18	No	28 days	1
Takehiro et al. (2014)	24	26			80–110	140–180	110	180			No	1 day	2
Ehab A. Wahby et al. (2016)	100	100	164.1 ± 12.1	167 ± 17	110–149	150–180					No	weaned from mechanical ventilation	2
Federico Bilotta et al. (2009)	10	10	179 ± 32	181 ± 29	80–110	180–215	110	215	92.3 ± 2.5	143.3 ± 20.3	Yes	6 months	6
Shalin P. Desai et al. (2012)	41	45			90–120	121–180					No	30 days	1
Federico Bilotta et al. (2007)	10	11			80–120	80–220					Yes	6 months	5
Michael SD Agus et al. (2012)	0	0			80–110		110		112(104–120)	121(109–136)	Yes	30 days	6
Harold L et al. (2011)	100	100	161 ± 50	151 ± 35	80–120	120–180	120	180	103 ± 17	135 ± 12	No	30 days	1

*TGC* tight glycemic control, *CGC* conventional glycemic control

### Outcomes definition and quality assessment

The primary endpoint for the current review was any postoperative infection including wound infection, pneumonia, urinary tract infection and sepsis. Secondary efficacy outcomes were the duration of mechanical ventilation, length of ICU stay, length of hospital stay (LOS) and other adverse events included the following: (1) short-term mortality (30-day mortality or hospital mortality). Any postoperative mortality including the following outcomes: 30-day mortality, hospital mortality, 6-month mortality and 1-year mortality; (2) neurological dysfunction including delirium, seizures and stroke; (3) acute renal failure that required postoperative CRRT (continuous renal replacement therapy); and (4) hypoglycemia, which was defined as a BG < 70 mg/dL, and the reported severe hypoglycemia (BG < 40 mg/dL).

### Quality of the included studies

The quality assessment of studies was independently performed by two investigators (Long CHEN and Fang TIAN) and the Jadad scale was used to assess the methodological quality of individual studies [11]. These evaluative criteria included the generation of allocation sequence (2 points), allocation concealment (2 points), investigator blindness (2 points), description of withdrawals and drop-outs (1 points), and the efficacy of randomization (2 points) (Additional file 2). The discrepancies were resolved by a third author.

### Data synthesis and analysis

The statistical analysis was performed using STATA (Version 12.0). Dichotomous data were expressed as the risk ratio (relative risk [RR]) with 95% confidence interval (CI). Data were pooled using a random-effects model (M-H heterogeneity). Statistical heterogeneity was tested by the *I*^2^ statistic and the χ2 test and heterogeneity was considered to be significant when values *I*^2^ > 50% and *p* ≤ 0.05. Subgroup analyses and meta-regression analyses were applied to detect the potential sources of heterogeneity. Sensitivity analysis was performed to assess the stability of the results. Publication bias was evaluated by Egger’s and Begg’s test, but it was not performed as the number of included studies was less than10 [12]. All reported *p* values were two-tailed and values of less than 0.05 were considered to be statistically significant.

### Sample size calculation and power analysis

Previous study showed a significant benefit for an intensive compared with a conventional glucose control protocol in reducing post-operative infection (OR 0·43, 0·29 to 0·64), but no significant benefit of death was found (OR 0·74, 0·45 to 1·23) [13]. A two-tailed power analysis with 0.80 power and an α of 0.05 was used. The post-operative infection and short-term mortality in CGC group was 15.8 and 5.4% respectively, with an estimated standard deviation of 249 patients were needed for post-operative infection and 4020 patients were needed for short-term mortality per study group. About 2233 patients per group were included in the study so the analysis was underpowered to identify an effect of TGC on mortality. Analysis was computed using GPower 3.1.9.2 Software.

## Results

### Study selection and characteristics

**Fig. 1:**
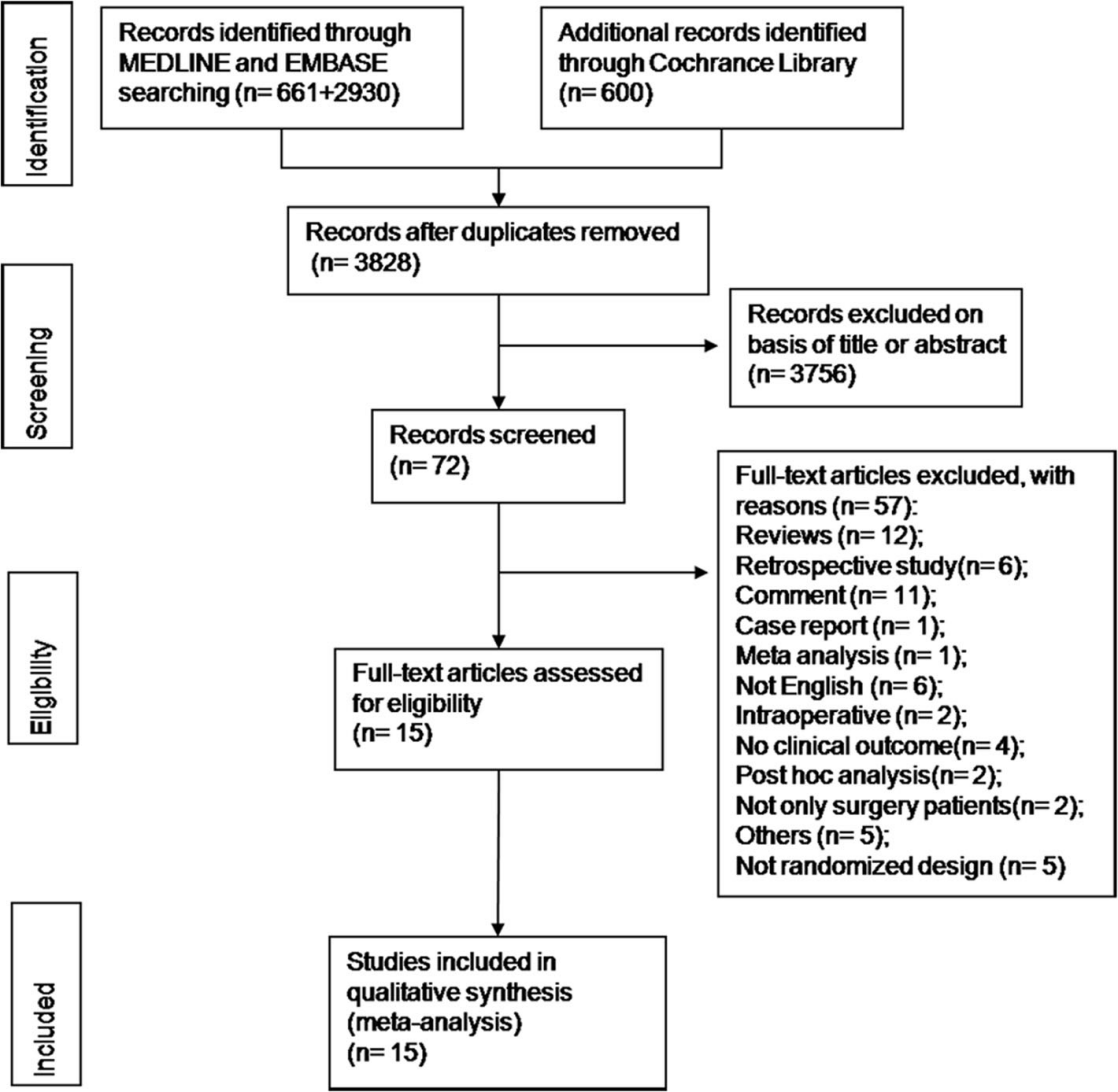
Flowchart of the study search, selection and inclusion process

Our search strategy identified 4191 articles. After screening the titles and abstracts, 4119 articles were excluded. The remaining 72 articles underwent a full-text review, although the study by van den Berghe et al. included adults receiving mechanical ventilation who were admitted to intensive care unit (which included mainly surgical patients (about 92%) and 62.5% had undergone cardiac surgery), because of the importance of this study, we still included it in our meta-analysis. Finally a total of 15 articles that had enrolled 5053 patients were finally included in this meta-analysis [1, 14–27] (Fig. 1). The detailed characteristics of these studies are presented in Table 1. About 1056 (20.9%) patients had diabetes, nine articles of included 15 articles mainly reported cardiac surgery including 3455 patients (68.4%). Among the included 15 articles, eight articles define tight glycemic control and trigger blood glucose as blood glucose ≤110 mg/dL, three articles were ≤ 120 mg/dL, the last four articles were ≤ 130 mg/dL, ≤140 mg/dL, ≤150 mg/dL and ≤ 160 mg/dL respectively. Eight articles only tight control the blood glucose postoperatively, the other seven articles were during intra and post operative period. The average Jadad Score of the studies included in the meta-analyses was 2.9, only five studies have exceeded 4.

### Primary outcomes

#### Risk of postoperative infection

**Fig. 2:**
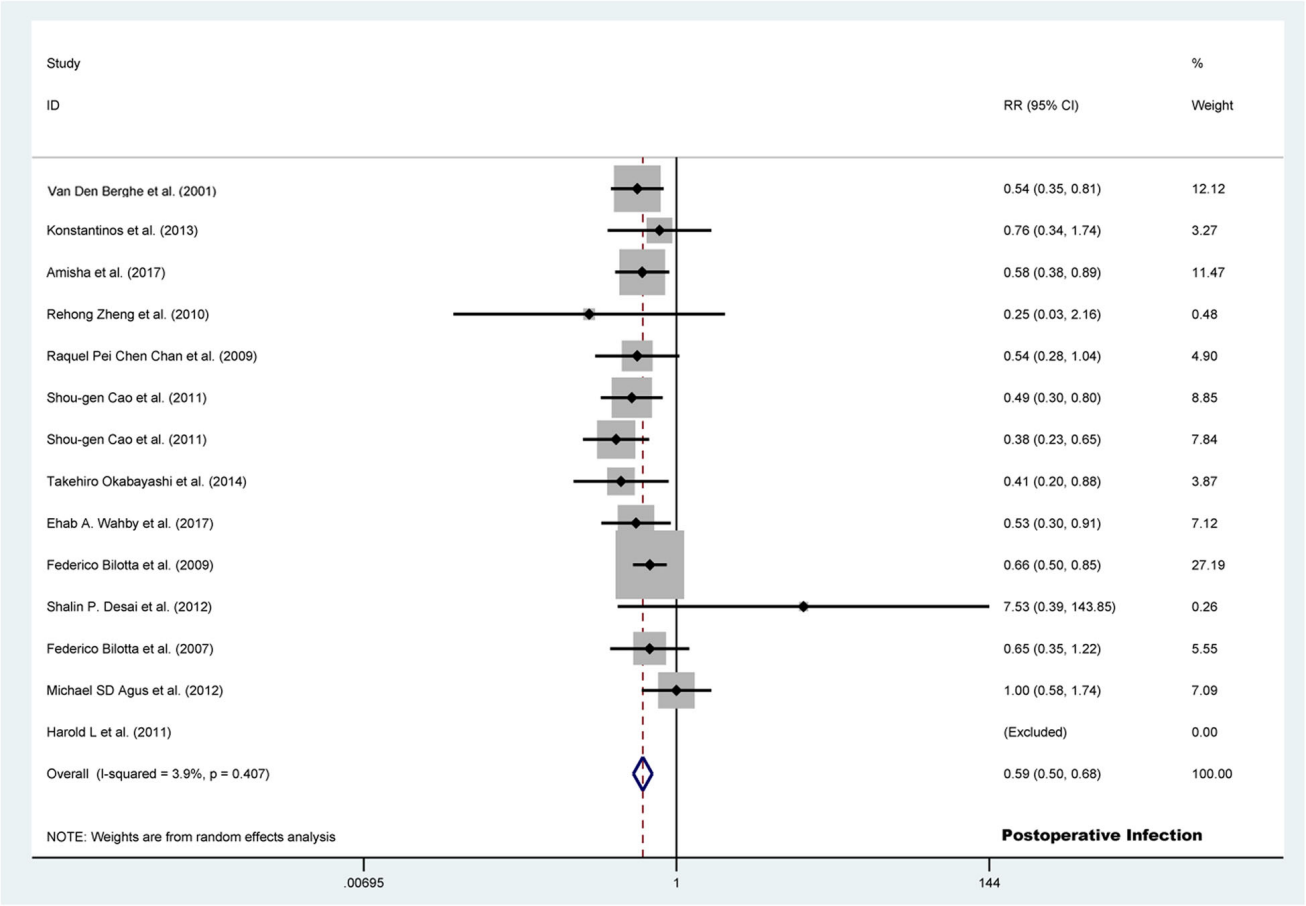
Forest plot of the risk of postoperative infection in TGC group versus control group. TGC = tight glycemic control; RR = relative risk; CI = confidence interval

Fourteen studies (*n* = 4952) compared the effectiveness of TGC versus CGC with respect to postoperative infection. These studies revealed that the risk of total postoperative infection (9.4% vs. 15.8%; RR 0.586, 95% CI 0.504 to 0.680, *p* <  0.001; Fig. 2) and sepsis (2.7% vs. 4.7%; RR 0.594, 95% CI 0.418 to 0.842, *p* = 0.003) were significantly lower in the TGC group than in the CGC group. The most frequent type of infection in the TGC group and CGC group was wound infection (4.6% vs. 7.2%; RR 0.620, 95% CI 0.422 to 1.910, *p* = 0.015). However, no difference was found in pneumonia (2.0% vs. 2.9%; RR 0.692, 95% CI 0.400 to 1.196, *p* = 0.187) and urinary tract infection (3.6% vs. 4.4%; RR 0.843, 95% CI 0.548 to 1.297, *p* = 0.437). In addition, there was no significant heterogeneity between articles (*I*^2^ < 50%, *p* > 0.05; Table 2).

**Table 2:** Postoperative adverse events and other outcomes

		TGC		CGC		Overall event rates (%)	M-H pooled RR		Heterogeneity	
Events	Studies	N+(%)	Total	N+(%)	Total		RR/ SMD (95%CI)	*p*	*I* ^**2**^ **(%)**	*p*
Infection	[1, 15–27]	231(9.4%)	2464	392(15.8%)	2488	12.6%	0.586(0.504, 0.680)	< 0.001	3.9	0.407
Sepsis	[1, 19, 20, 23, 25, 26]	48(2.7%)	1753	82(4.7%)	1763	3.7%	0.594(0.418, 0.842)	0.003	< 0.001	0.945
Pneumonia	[19, 20, 23–26]	22(2.0%)	1079	31(2.9%)	1078	2.5%	0.692(0.400, 1.196)	0.187	< 0.001	0.511
Urinary tract infection	[19, 20, 23, 25, 30]	36(3.6%)	988	43(4.4%)	980	4.0%	0.843(0.548, 1.297)	0.437	< 0.001	0.682
Wound infection	[19, 20, 22–27]	54(4.6%)	1186	86(7.2%)	1188	6.1%	0.620(0.422, 0.910)	0.015	18.8	0.287
Short-term mortality	[1, 14–22, 24, 27]	85(3.8%)	2233	122(5.4%)	2259	4.6%	0.692(0.527, 0.909)	0.008	< 0.001	0.769
Any mortality	[1, 14–27]	159(6.3%)	2514	204(8.0%)	2539	7.2%	0.792(0.653, 0.960)	0.018	< 0.001	0.738
Neurological dysfunction	[14, 18, 22, 24, 26]	8(1.1%)	752	18(2.4%)	761	1.7%	0.499(0.219, 1.137)	0.098	< 0.001	0.651
Acute renal failure	[1, 22, 24, 26]	46(3.3%)	1413	78(5.4%)	1439	4.3%	0.610(0.359, 1.038)	0.068	16.0	0.312
Hypoglycemia	[1, 16–20, 22–24, 27]	467(22.3%)	2097	233(11.0%)	2118	16.6%	3.145(1.928, 5.131)	< 0.001	81.4	< 0.001
Severe hypoglycemia	[15, 16, 19–21, 24, 26]	34(2.8%)	1207	8(0.7%)	1210	1.7%	3.821(1.796, 8.127)	< 0.001	< 0.001	0.894
ICU stay	[1, 15, 16, 18, 23, 26, 27]						−0.428(−0.833, −0.022)	0.039	96.6	< 0.001
Mechanical ventilation	[1, 15, 16, 18, 23, 26, 27]						−0.275(−0.695,0.146)	0.201	96.9	< 0.001
LOS	[15, 16, 18–20, 26, 27]						−0.233(−0.496,0.030)	0.082	85.4	< 0.001

*N*^*+*^ the number of patient with adverse event, *Total* the number of the total patients, *RR* relative risk, *SMD* standardised mean difference, *LOS* length of hospital stay

### Sensitivity analysis and publication Bias

The results of sensitivity analyses showed consistency in the results with the omission of a single article per replication (Additional file 3: Table S1). A funnel plot of the risk of postoperative infection identified all studies in the 95% confidence limits (Additional file 4: Figure S1).

#### Risk of postoperative short-term mortality

**Fig. 3:**
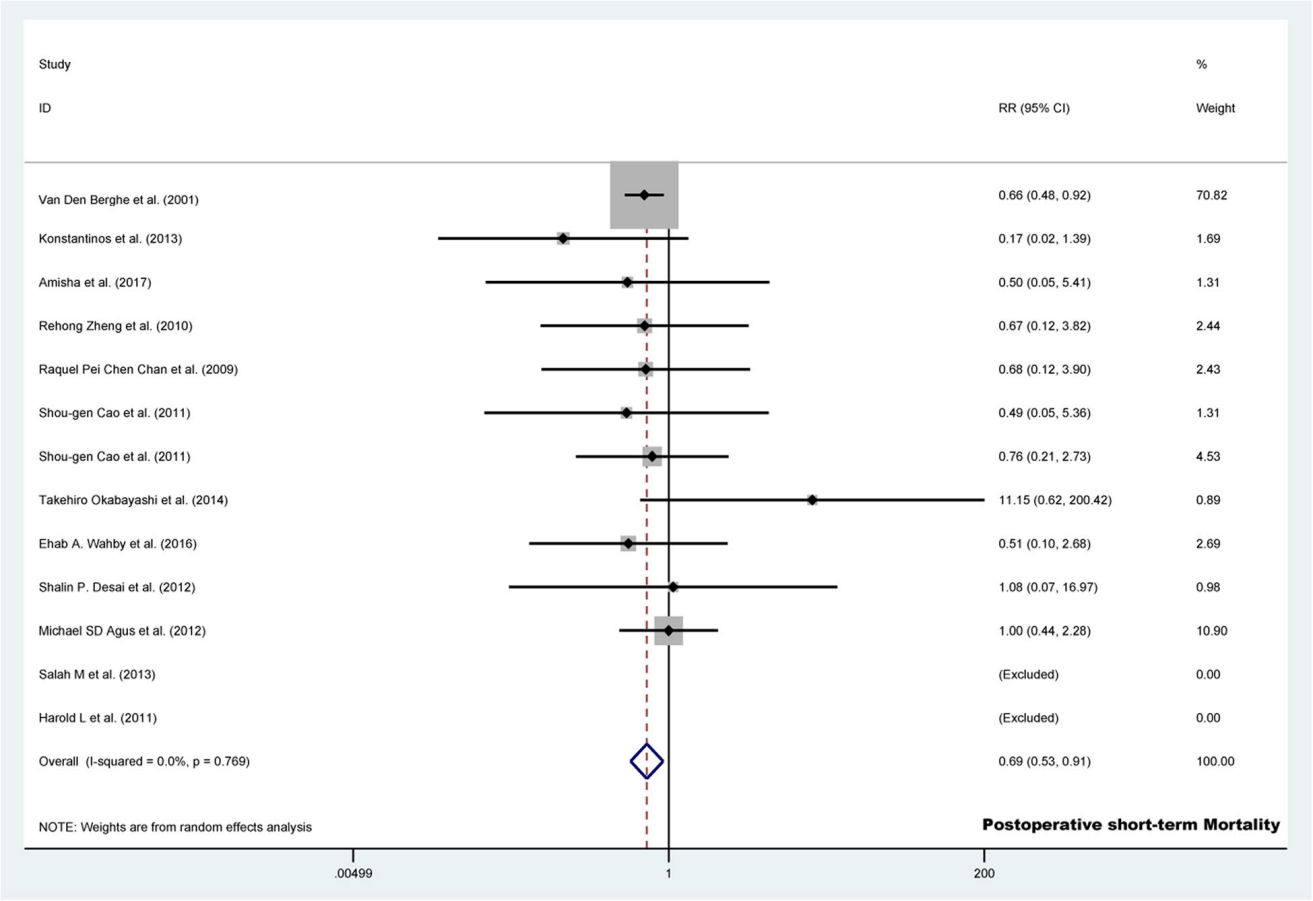
Forest plot of the risk of postoperative short-term mortality in TGC group versus control group. TGC = tight glycemic control; RR = relative risk; CI = confidence interval

Of the 15 studies included, 13 studies (*n* = 4492) compared the effectiveness of TGC versus CGC to assess the risk of postoperative short-term mortality. TGC showed a lower risk of postoperative short-term mortality (3.8% vs. 5.4%; RR 0.692, 95% CI 0.527 to 0.909, *p* = 0.008; Fig. 3) and any postoperative mortality (6.3% vs. 8.0%; RR 0.792, 95% CI 0.653 to 0.960, *p* = 0.018; Additional file 5: Figure S2) without evidence of heterogeneity between articles (*I*^2^ <  0.001%, *p* > 0.05; Table 2).

### Sensitivity analysis and publication Bias

Sensitivity analyses showed that the result was mainly influenced by the study of van den Berghe when eliminating one single study per replication (Additional file 6: Table S2). A funnel plot of the risk of postoperative short-term mortality revealed that no included studies exceeded the 95% confidence limits (Additional file 7: Figure S3). Begg’s (*p* = 0.533 for short-term mortality and *p* = 0.951 for any postoperative mortality; Additional file 8: Figure S4A) and Egger’s test (*p* = 0.741 for short-term mortality and *p* = 0.814 for any postoperative mortality; Additional file 8: Figure S4B) did not identify significant publication bias.

#### Risk of postoperative neurological dysfunction and acute renal failure

Only five (*n* = 1513) and four (*n* = 2852) articles reported postoperative neurological dysfunction and postoperative acute renal failure respectively. No significant difference was observed in postoperative neurological dysfunction (1.1% vs. 2.4%; RR 0.499, 95% CI 0.219 to 1.137, *p* = 0.098) and postoperative acute renal failure (3.3% vs. 5.4%; RR 0.610, 95% CI 0.359 to 1.038, *p* = 0.068), and no significant heterogeneity was observed between articles (*I*^2^ < 50%, *p* > 0.05; Table 2). In addition, sensitivity analyses revealed a consistency of the results based on the omission of a single article at a time for acute renal failure, but not for neurological dysfunction (Additional file 9: Table S3, Additional file 10: Table S4). Publication bias analysis was not performed as the number of included studies was less than10 [12].

#### Risk of postoperative hypoglycemia

**Fig. 4:**
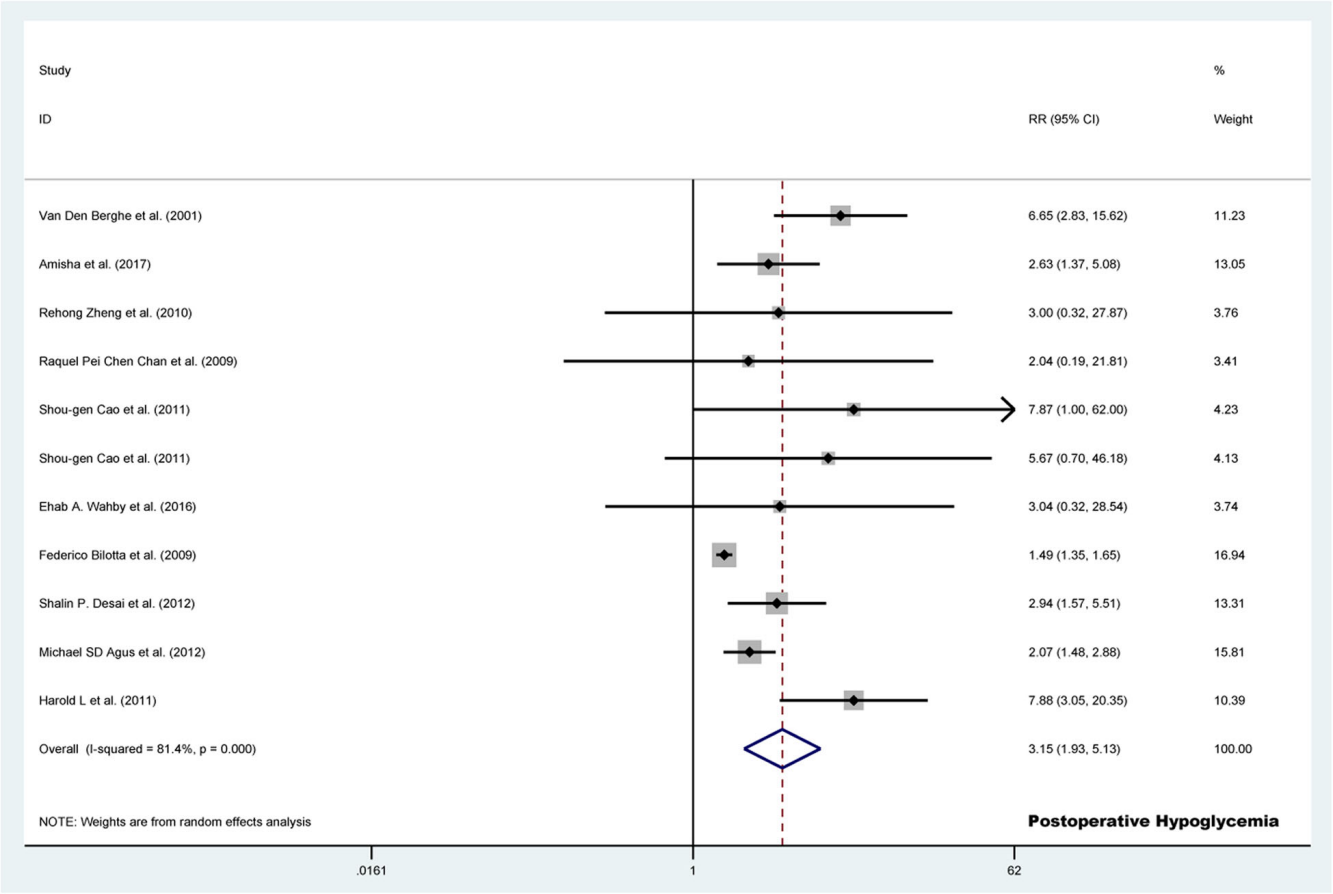
Forest plot of the risk of postoperative hypoglycemia in TGC group versus control group. TGC = tight glycemic control; RR = relative risk; CI = confidence interval

Eleven (*n* = 4215) studies compared the safety of TGC versus CGC to assess the risk of postoperative hypoglycemia. We observed more patients experiencing postoperative hypoglycemia (22.3% vs. 11.0%; RR 3.145, 95% CI 1.928 to 5.131, *p* <  0.001; Fig. 4) and severe hypoglycemia (2.8% vs. 0.7%; RR 3.821, 95% CI 1.796 to 8.127, *p* <  0.001; Additional file 11: Figure S5) in the TGC group as compared to the CGC group. No significant heterogeneity was observed respect to severe hypoglycemia (*I*^2^ <  0.001%, *p* = 0.894), however, there was significant heterogeneity between articles with a corresponding *I*^2^ of 81.4% (*p* <  0.001) with respect to postoperative hypoglycemia (Table 2).

### Sensitivity analysis and publication Bias

Sensitivity analyses revealed that there was no significant heterogeneity of postoperative hypoglycemia (*I*^2^ = 33.9%, *p* = 0.137 Additional file 12: Table S5) when we omitted Federico Bilotta’s study of neurosurgical patients. The result was consisted with a significant higher rate of postoperative hypoglycemia in the TGC groups (13.0% vs. 4.3%; RR 3.361, 95% CI 2.311 to 4.890, *p* <  0.001; Additional file 12: Table S5). A funnel plot of the risk of postoperative hypoglycemia identified two studies beyond the 95% confidence limits (Additional file 13: Figure S6).

Sensitivity analyses showed consistency in the results of severe hypoglycemia with the omission of a single article per replication (Additional file 14: Table S6).

### Subgroup analysis and meta-regression of postoperative hypoglycemia

Then, subgroup analyses and meta-regression were further applied to examine the sources of the heterogeneity. In the subgroup analyses, we found that the type of surgery, but not preoperative diabetes, the type of patient, the time of intervention, the trigger of blood glucose level, and the use of glucocorticoids in the hospital, could explain the heterogeneity (Additional file 15: Table S7).

However, the result of meta-regression could not identify that the type of surgery was the source of the heterogeneity. The use of glucocorticoids in the hospital was seem to be the source of the observed heterogeneity, but factors such as preoperative diabetes, the type of patient, the time of intervention, trigger of blood glucose level, the mean age, the sample size and the quality of the study did not seem to be the source of the observed heterogeneity (Additional file 16: Table S8).

#### Length of ICU stay and hospitalization

About half of the included studies reported duration of mechanical ventilation, length of ICU stay and hospitalization. TGC showed less length of ICU stay (SMD, − 0.428 days; 95% CI, − 0.833 to − 0.022 days; *p* = 0.039, Additional file 17: Figure S7), but TGC showed neutral effect on postoperative duration of mechanical ventilation (SMD, − 0.275 h; 95% CI, − 0.695 to 0.146 h; *p* = 0.201, Additional file 18: Figure S8) and length of hospitalization (SMD, − 0.233 days; 95% CI, − 0.496 to 0.030 days; *p* = 0.082, Additional file 19: Figure S9) (Table 2). In the sensitivity analysis, we found significant heterogeneity between the studies (Additional file 20: Table S9, Additional file 21: Table S10, Additional file 22: Table S11).

### Subgroup analysis and meta-regression of ICU stay and hospitalization

The results of the subgroup analyses and meta-regression analyses revealed that the type of surgery, preoperative diabetes, type of patient, time of intervention, trigger of blood glucose level and use of glucocorticoids in hospital, the year of publication, the mean age, the sample size, and the quality of the study could not identify the heterogeneity; thus, heterogeneity persisted between the included articles (Additional file 23: Table S12, Additional file 24: Table S13, Additional file 25: Table S14, Additional file 26: Table S15, Additional file 27: Table S16, Additional file 28: Table S17).

## Discussion

In the current meta-analysis of randomized trials, we found that when compared to CGC, TGC immediately after surgery significantly reduces total postoperative infection rates and short-term mortality. However, it might limit conclusion regarding the efficacy of TGC for short-term mortality in sensitivity analyses. The patients in the TGC group experienced a significant higher rate of postoperative hypoglycemia. TGC had a neutral effect on the risk of postoperative neurological dysfunction and acute renal failure in patients undergoing surgery. Although there was significant heterogeneity of hypoglycemia between studies, which was primarily caused by the study of Federico Bilotta et al., the pooled RR still derived the same results after omitting this study.

There was still some controversy on the positive effects of TGC in reducing postoperative infection. Some meta-analysis found that intra-operative TGC [28] or using a TGC strategy in the perioperative period (< 150 mg/dl) [13] decreased the infection rate when compared to the conventional therapy; however, other meta-analyses have reported negative effects [29, 30]. Furthermore, most of those studies just focused on cardiac surgery. A recent retrospective analysis [31] found that the basal + premeal insulin regimen was associated with a reduced rate of postoperative infective complications than the premeal insulin alone therapy, without increasing the number of severe hypoglycemic events. These results suggest that type of treatments more than levels of glycemic controls might have on beneficial effect these outcomes. This meta-analysis has shown that TGC significantly reduced total postoperative infection, wound infection and sepsis regardless of whether TGC was commenced during or after surgery, but no difference was found in pneumonia and urinary tract infection. This study may suggest that TGC should be administrated especially in those with high postoperative infection risk.

Our meta-analysis further supported the study of van den Berghe [1] that perioperative TGC reduce the rates of postoperative short-term mortality, but sensitivity analyses showed a negative result when eliminating the study of van den Berghe. It might limit conclusion regarding the efficacy of TGC for mortality. Another meta-analysis [32] also found that moderate perioperative glycemic control (BG 150–200 mg/dL) was associated with lower postoperative mortality and stroke in patients with diabetes, whereas no additional benefit was found in a stricter glycemic control group (BG < 150 mg/dL). We should also note that this meta-analysis not only includes randomized studies, but also retrospective studies. A recent retrospective study analyzed a large database of patients in critical care units [33] and found that TGC (80–110 mg/dL) was associated with the lowest mortality. However, previous meta-analysis found that perioperative TGC did not reduce the rates of short-term mortality in ICU settings [29] or in various hospital settings [28, 34] when compared to the conventional therapy. In the NICE-SUGAR trial (Normoglycemia in Intensive Care Evaluation-Survival Using Glucose Algorithm Regulation trial), 6104 critically ill patients in intensive care units (ICUs) were randomized to an intensive blood glucose control (81–108 mg/dL) or conventional glucose control (< 180 mg/dL) group. It showed that TGC actually increased 90-day mortality and hypoglycemia compared to a more liberal glucose target in the ICU setting [2, 3]. Recently, in the GLUCO-CABG trial [5], CABG patients were randomized to TGC (100 to 140 mg/dL) and CGC (141 to 180 mg/dL) groups just in the ICU. The two groups had no significant difference in complications rates of mortality, wound infection, acute kidney injury, or other outcomes, and in the incidence of hypoglycemia or length of hospital stay.

Most studies [8, 9] and meta-analysis [29, 32] found a neutral effect of TGC on other clinical outcomes including neurological dysfunction, acute renal failure and length of hospital stay; our result further supported those findings. Our finding was not consistent with the clinical practice guideline from the American College of Physicians [30] that they found TGC was not associated with a reduction of ICU stay in the mixed medical intensive care unit/surgical intensive care unit environment. Another recent study [9] found that only nondiabetic cardiac surgery patients, but not patients with diabetes, could gain significant benefit of postoperative complications from intraoperative TGC.

The major harm of TGC was that it might increase hypoglycemia, especially in critically ill patients [2, 35, 36]. The study by Finfer S et al. found that intensive glucose control leads to moderate and severe hypoglycemia in critically ill patients [2]. Our finding was also consistent with many studies [13] and other meta-analysis [29, 34], which have also noted that using a TGC protocol in the perioperative period increased the risk of hypoglycemia, but without a significant increase in serious adverse events in various hospital settings. However, a recent meta-analysis [28] that found intraoperative insulin therapy may not increase the rate of hypoglycemia. The consequences of hypoglycemia in hospitalized patients remain unclear as few studies report clinical adverse effects and explain how hypoglycemia harms patients in the long-term consequences. The study of Finfer S [2] has confirmed that both of moderate and severe hypoglycemia was associated with an increased risk of death in critically ill patients, however, others argued [37] that hyperglycemia was more similar to a signal of illness severity rather than the cause of clinical adverse outcomes. Indeed, the hyperglycemia level was related to the activation of the stress response.

The conflict of the results between the present meta-analysis and others can be explained by different inclusion criteria, patient characteristics, time of TGC, type of treatments, hospital setting, and the definition of hypoglycemia. Furthermore, the markedly variation in blood glucose target levels, the protocols of glucose monitoring and managing among studies may also influence the results.

There are some strengths of this study. For the first time, the present meta-analysis was conducted to evaluate the association between postoperative glycemic control and outcomes in patients undergoing elective surgery. Second, we have included the most rigorous analysis of TGC studies to date and conducted a comprehensive meta-analysis to elevate the effect of postoperative TGC on outcomes. However, high-quality evidence to support the routine use of postoperative TGC is still lacking.

### Limitations

There are some limitations in this meta-analysis. First, the source data were extracted from diverse types of surgery and glycemic targets. In addition, there were variations in the timing of the intervention (postoperative versus intra-operative plus post-operative). Finally, the number of eligible studies was small; thus the results were likely biased. This may underestimate the benefit of TGC. Despite these differences, no significant heterogeneity was observed between studies with respect to the primary endpoint and most other outcomes and our results were consistent in the sensitivity analyses.

## Conclusions

The results of this study show that TGC immediately after surgery significantly reduces total postoperative infection rates and short-term mortality. However, it might limit conclusion regarding the efficacy of TGC for short-term mortality in sensitivity analyses. The patients in the TGC group experienced a significant higher rate of postoperative hypoglycemia. TGC had a neutral effect on the risk of postoperative neurological dysfunction and acute renal failure as compared to CGC. This study may suggest that TGC should be administrated under close glucose monitoring in patients undergoing surgery, especially in those with high postoperative infection risk. In addition, large, prospective, randomized and high quality trials on the efficacy and safety of TGC in the postoperative period are needed to investigate the ideal BG target to optimize clinical outcomes and minimize adverse events in patients undergoing surgery.

## Additional files

Additional file 1: Search strategies for this study. (DOC 38 kb)

Additional file 2: The Jadad scale for assessing the methodological quality of clinical trials. (DOC 27 kb)

Additional file 3: **Table S1**. Sensitivity analysis for the outcome of the risk of postoperative infection. (DOC 49 kb)

Additional file 4: **Figure S1**. A funnel plot of the risk of postoperative infection. (TIF 301 kb)

Additional file 5: **Figure S2.** Forest plot of the risk of any postoperative mortality in TGC group versus control group. TGC = tight glycemic control; RR = relative risk; CI = confidence interval. (TIF 445 kb)

Additional file 6: **Table S2**. Sensitivity analysis for the outcome of the risk of postoperative short-term mortality. (DOC 48 kb)

Additional file 7: **Figure S3**. A funnel plot of the risk of postoperative short-term mortality. (TIF 299 kb)

Additional file 8: **Figure S4.** A: Begg’s test for short-term mortality; B: Egger’s test for short-term mortality. (TIF 365 kb)

Additional file 9: **Table S3**. Sensitivity analysis for the outcome of the risk of postoperative neurological dysfunction. (DOC 38 kb)

Additional file 10: **Table S4**. Sensitivity analysis for the outcome of the risk of postoperative acute renal failure. (DOC 35 kb)

Additional file 11: **Figure S5**. Forest plot of the risk of postoperative servese hypoglycemia in TGC group versus control group. TGC = tight glycemic control; RR = relative risk; CI = confidence interval. (TIF 300 kb)

Additional file 12: **Table S5**. Sensitivity analysis for the outcome of the risk of postoperative hypoglycemia. (DOC 47 kb)

Additional file 13: **Figure S6**. A funnel plot of the risk of postoperative hypoglycemia. (TIF 293 kb)

Additional file 14: **Table S6**. Sensitivity analysis for the outcome of the risk of postoperative servese hypoglycemia. (DOC 40 kb)

Additional file 15: **Table S7.** Subgroup analyses for the outcome of the risk of postoperative hypoglycemia. (DOC 68 kb)

Additional file 16: **Table S8**. Meta-regression for the outcome of the risk of postoperative hypoglycemia. (DOC 44 kb)

Additional file 17: **Figure S7**. Forest plot of the risk of postoperative ICU stay in TGC group versus control group. TGC = tight glycemic control; SMD = standardised mean difference; CI = confidence interval . (TIF 296 kb)

Additional file 18: **Figure S8**. Forest plot of the risk of postoperative duration of mechanical ventilation in TGC group versus control group. TGC = tight glycemic control; SMD = standardised mean difference; CI = confidence interval. (TIF 300 kb)

Additional file 19: **Figure S9**. Forest plot of the risk of postoperative LOS in TGC group versus control group. TGC = tight glycemic control; SMD = standardised mean difference; CI = confidence interval; LOS = length of hospitalization. (TIF 297 kb)

Additional file 20: **Table S9**. Sensitivity analysis for the outcome of the risk of postoperative ICU stay. (DOC 40 kb)

Additional file 21: **Table S10**. Sensitivity analysis for the outcome of the risk of postoperative duration of mechanical ventilation. (DOC 40 kb)

Additional file 22: **Table S11.** Sensitivity analysis for the outcome of the risk of postoperative length of hospitalization. (DOC 41 kb)

Additional file 23: **Table S12.** Subgroup analyses for the outcome of the risk of postoperative ICU stay. (DOC 55 kb)

Additional file 24: **Table S13.** Meta-regression for the outcome of the risk of postoperative ICU stay. (DOC 45 kb)

Additional file 25: **Table S14**. Subgroup analyses for the outcome of the risk of postoperative duration of mechanical ventilation. (DOC 55 kb)

Additional file 26: **Table S15**. Meta-regression for the outcome of the risk of postoperative duration of mechanical ventilation. (DOC 44 kb)

Additional file 27: **Table S16**. Subgroup analyses for the outcome of the risk of postoperative length of hospitalization. (DOC 60 kb)

Additional file 28: **Table S17.** Meta-regression for the outcome of the risk of postoperative length of hospitalization. (DOC 45 kb)

## Abbreviations

BG: Blood glucose level
CGC: Conventional glycemic control
CI: Confidence interval
CRRT: Continuous renal replacement therapy
ICU: Intensive care unit
LOS: Length of hospital stay
RR: Relative risk
SMD: Standardised mean difference
TGC: Tight glycemic control
