# Supplementary material for: Postoperative tight glycemic control significantly reduces postoperative infection rates in patients undergoing surgery: a meta-analysis
Source: BMC Endocr Disord. 2018 Jun 22;18:42. doi: 10.1186/s12902-018-0268-9 (PMC6013895; doi:10.1186/s12902-018-0268-9)
Supplement: Supplementary file 1 — Search strategies for this study. (DOC 38 kb) [file 12902_2018_268_MOESM1_ESM.doc]

**Appendix 1**

**Database**

Cochrane Central Register of Controlled Trials (CENTRAL) in the Cochrane Library

**Searchfilter**

#1 blood glucose [mh]

#2 insulin [mh]

#3 Postoperative Period [mh]

#4 Blood Sugar

#5 Sugar, Blood

#6 Glucose, Blood

#7 glucose blood level

#8 blood glucose monitoring

#9 glycemic control

#10 Insulin, Regular

#11 Regular Insulin

#12 Soluble Insulin

#13 Insulin, Soluble

#14 Insulin A Chain

#15 Sodium Insulin

#16 Insulin, Sodium

#17 Novolin

#18 Iletin

#19 Insulin B Chain

#20 Chain, Insulin B
#21 Period, Postoperative

#22 Periods Postoperative

#23 Postoperative Periods

#24 #1 OR #4 OR #5 OR #6 OR #7 OR #8 OR #9

#25 #2 OR #10 OR #11 OR #12 OR #13 OR #14 OR #15 OR #16 OR #17 OR #18 OR #19 OR #20

#26 #24 OR #25

#27 #3 OR #21 OR #22 OR #23

#28 #26 AND #27

**Database**

Medline (Pubmed)

**Searchfilter**

(blood glucose [mh] OR Blood Sugar[Title/Abstract] OR Sugar, Blood[Title/Abstract] OR Glucose, Blood[Title/Abstract] OR glucose blood level[Title/Abstract] OR blood glucose monitoring[Title/Abstract] OR glycemic control[Title/Abstract]) OR (insulin [mh] OR Insulin, Regular[Title/Abstract] OR Regular Insulin[Title/Abstract] OR Soluble Insulin[Title/Abstract] OR Insulin, Soluble[Title/Abstract] OR Insulin A Chain[Title/Abstract] OR Sodium Insulin[Title/Abstract] OR Insulin, Sodium[Title/Abstract] OR Novolin[Title/Abstract] OR Iletin[Title/Abstract] OR Insulin B Chain[Title/Abstract] OR Chain, Insulin B[Title/Abstract]) AND (Postoperative Period [mh] OR Period, Postoperative OR Periods Postoperative OR Postoperative Periods) AND (randomized controlled trial[pt] OR controlled clinical trial[pt] OR randomized[tiab] OR placebo[tiab] OR drug therapy[sh] OR randomly[tiab] OR trial[tiab] OR groups[tiab])

**Database**

EMBASE (Ovid SP)

**Searchfilter**

#1 'glucose blood level'/exp
#2 'blood glucose':ab,ti OR 'blood sugar':ab,ti OR 'sugar, blood':ab,ti OR 'glucose, blood':ab,ti OR 'blood glucose monitoring':ab,ti OR 'glycemic control':ab,ti
#3 #1 OR #2
#4 'insulin'/exp
#5 'insulin, regular':ab,ti OR 'regular insulin':ab,ti OR 'soluble insulin':ab,ti OR 'insulin, soluble':ab,ti OR 'insulin a chain':ab,ti OR 'sodium insulin':ab,ti OR 'insulin, sodium':ab,ti OR novolin:ab,ti OR iletin:ab,ti OR 'insulin b chain':ab,ti OR 'chain, insulin b':ab,ti
#6 #4 OR #5

#7 #3 OR #6

#8 'postoperative period'/exp

#9 'Period, Postoperative':ab,ti OR 'Periods Postoperative':ab,ti OR 'Postoperative Periods':ab,ti

#10 #8 OR #9

#11'randomized controlled trial'/exp OR 'controlled clinical trial'/exp OR randomized:ti,ab OR placebo:ti,ab OR 'drug therapy':lnk OR randomly:ti,ab OR trial:ti,ab OR groups:ti,ab

#12 #7 AND #10 AND #11
