# Supplementary material for: Postoperative tight glycemic control significantly reduces postoperative infection rates in patients undergoing surgery: a meta-analysis
Source: BMC Endocr Disord. 2018 Jun 22;18:42. doi: 10.1186/s12902-018-0268-9 (PMC6013895; doi:10.1186/s12902-018-0268-9)
Supplement: Supplementary file 2 — The Jadad scale for assessing the methodological quality of clinical trials. (DOC 27 kb) [file 12902_2018_268_MOESM2_ESM.doc]

**Appendix B: Jadad scale**

A clinical trial could receive a Jadad score of between zero and seven. 1-3 points were considered as low quality and 4-7 points were considered as high quality. The Jadad scale is concise and has only four questions which are as follows:

1. Generation of allocation sequence

A. The method to generate the sequence of randomization was described and it was appropriate (table of random numbers, computer generated, etc.). (+2 Points)

B. The study was described as randomized, but the method to generate the sequence of randomization was not described. (+1 Point)

C. The method to generate the sequence of randomization was described and it was inappropriate (patients were allocated alternately, or according to date of birth, hospital number, etc.). (+0 Point)

2. Allocation concealment

A. central randomization or sealed envelopes or similar. (+2 Points)

B. The study was described as randomized, but the method to allocation concealment was not described. (+1 Point)

C. Allocation concealment was not described or it was inappropriate (patients were allocated alternately, or according to date of birth, hospital number, etc.). (+0 Point)

3. Investigator blindness

A. The method of double blinding was described and it was appropriate (identical placebo, active placebo, dummy, etc.). (+2 Points)

B. The study was described as double blind, but the method of blinding was not described. (+1 Point)

C. The study was described as double blind but the method of blinding was inappropriate (e.g., comparison of tablet vs. injection with no double dummy). (+0 Point)

4. Was there a description of withdrawals and dropouts?

A. Yes. The number of withdrawals and dropouts and the reasons were stated in each of the comparison groups. (+1 Point)

B. No. (+0 Point)
