# Supplementary material for: Postoperative tight glycemic control significantly reduces postoperative infection rates in patients undergoing surgery: a meta-analysis
Source: BMC Endocr Disord. 2018 Jun 22;18:42. doi: 10.1186/s12902-018-0268-9 (PMC6013895; doi:10.1186/s12902-018-0268-9)
Supplement: Supplementary file 3 — Table S1. Sensitivity analysis for the outcome of the risk of postoperative infection. (DOC 49 kb) [file 12902_2018_268_MOESM3_ESM.doc]

**Supplemental table 1. Sensitivity analysisfor the outcome of the risk of any postoperative infection**

| **Study omitted** | **Estimate RR** | **95% CI** | | ***P* value** | **Heterogeneity** |  |
| --- | --- | --- | --- | --- | --- | --- |
|  |  | **Lower** | **Upper** |  | **I^2^ (%)** | ***P* value** |
| Van Den Berghe et al. (2001) | 0.590 | 0.497 | 0.700 | < 0.001 | 10.5 | 0.342 |
| Konstantinos et al. (2013) | 0.578 | 0.493 | 0.678 | < 0.001 | 9.0 | 0.358 |
| Amisha et al. (2017) | 0.583 | 0.491 | 0.693 | < 0.001 | 11.9 | 0.328 |
| Rehong Zheng et al. (2010) | 0.587 | 0.503 | 0.685 | < 0.001 | 7.4 | 0.372 |
| Raquel Pei Chen Chan et al. (2009) | 0.586 | 0.497 | 0.691 | < 0.001 | 11.4 | 0.333 |
| Shou-gen Cao et al. (2011) | 0.594 | 0.505 | 0.700 | < 0.001 | 7.9 | 0.368 |
| Shou-gen Cao et al. (2011) | 0.608 | 0.524 | 0.707 | < 0.001 | < 0.001 | 0.555 |
| Takehiro Okabayashi et al. (2014) | 0.594 | 0.508 | 0.693 | < 0.001 | 5.5 | 0.391 |
| Ehab A. Wahby et al. (2016) | 0.588 | 0.498 | 0.694 | < 0.001 | 10.7 | 0.340 |
| Federico Bilotta et al. (2009) | 0.562 | 0.470 | 0.671 | < 0.001 | 5.0 | 0.396 |
| Shalin P. Desai et al. (2012) | 0.584 | 0.505 | 0.675 | < 0.001 | < 0.001 | 0.565 |
| Federico Bilotta et al. (2007) | 0.579 | 0.491 | 0.683 | < 0.001 | 11.1 | 0.336 |
| Michael SD Agus et al. (2012) | 0.565 | 0.487 | 0.656 | < 0.001 | < 0.001 | 0.653 |
| Harold L et al. (2011) | 0.586 | 0.504 | 0.680 | < 0.001 | 3.9 | 0.407 |
| Combined | 0.586 | 0.504 | 0.680 | < 0.001 | 3.9 | 0.407 |

RR, Relative risk; CI, Confidence interval.
