# Supplementary figures and images for: Postoperative tight glycemic control significantly reduces postoperative infection rates in patients undergoing surgery: a meta-analysis
Source: BMC Endocr Disord. 2018 Jun 22;18:42. doi: 10.1186/s12902-018-0268-9 (PMC6013895; doi:10.1186/s12902-018-0268-9)

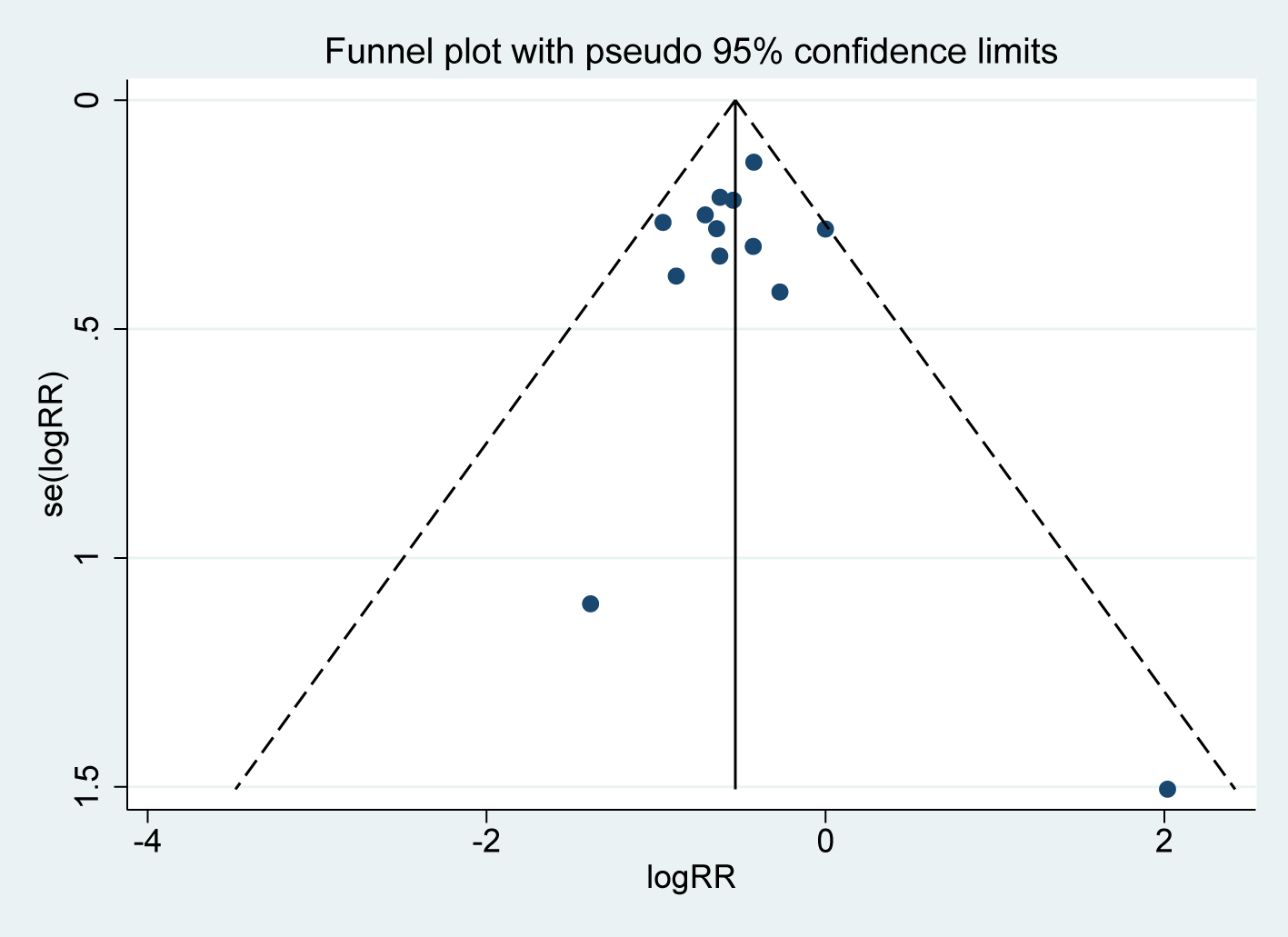

Supplement: Supplementary file 4 — Figure S1. A funnel plot of the risk of postoperative infection. (TIF 301 kb) [file 12902_2018_268_MOESM4_ESM.tif]

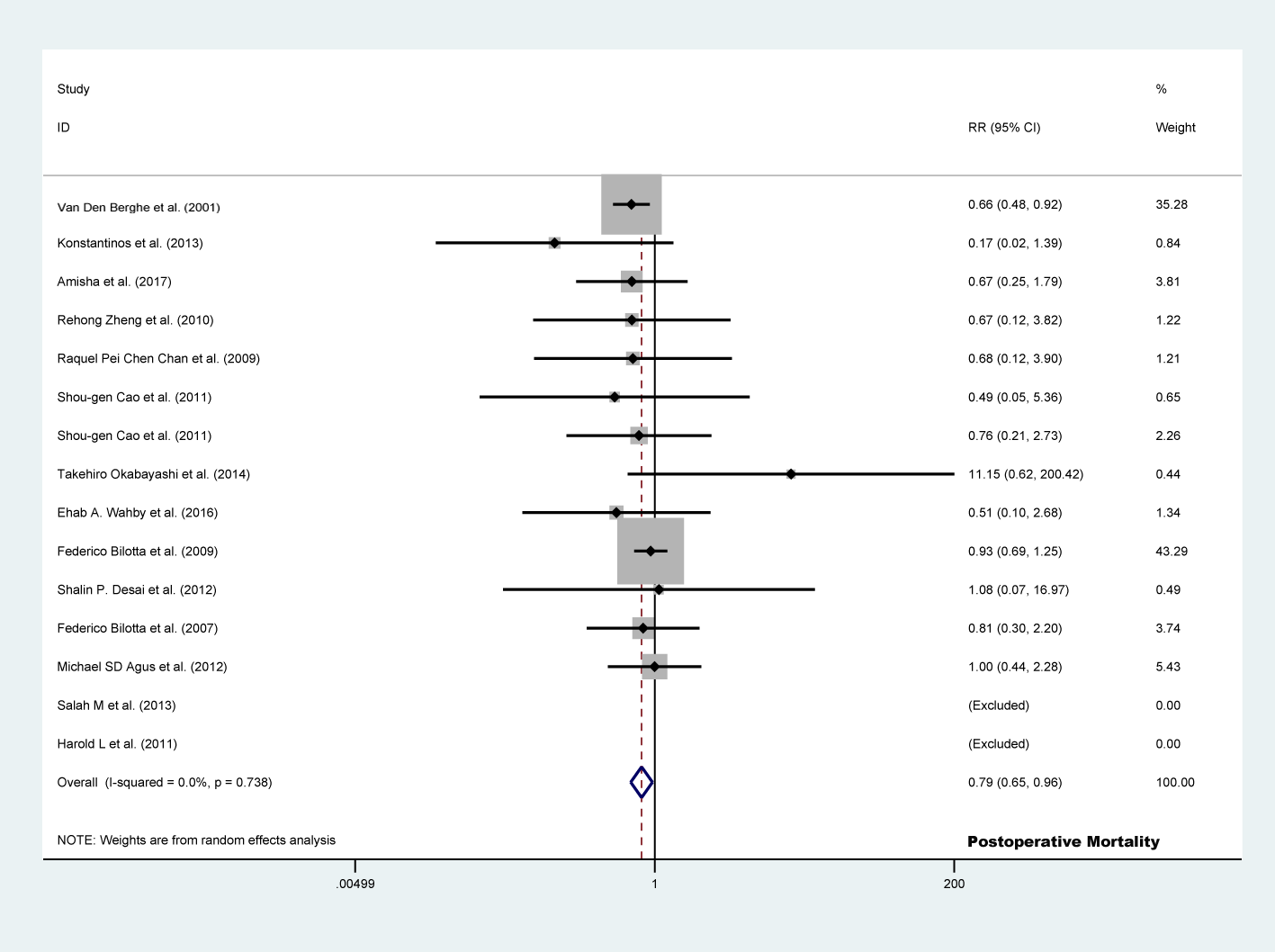

Supplement: Supplementary file 5 — Figure S2. Forest plot of the risk of any postoperative mortality in TGC group versus control group. TGC = tight glycemic control; RR = relative risk; CI = confidence interval. (TIF 445 kb) [file 12902_2018_268_MOESM5_ESM.tif]

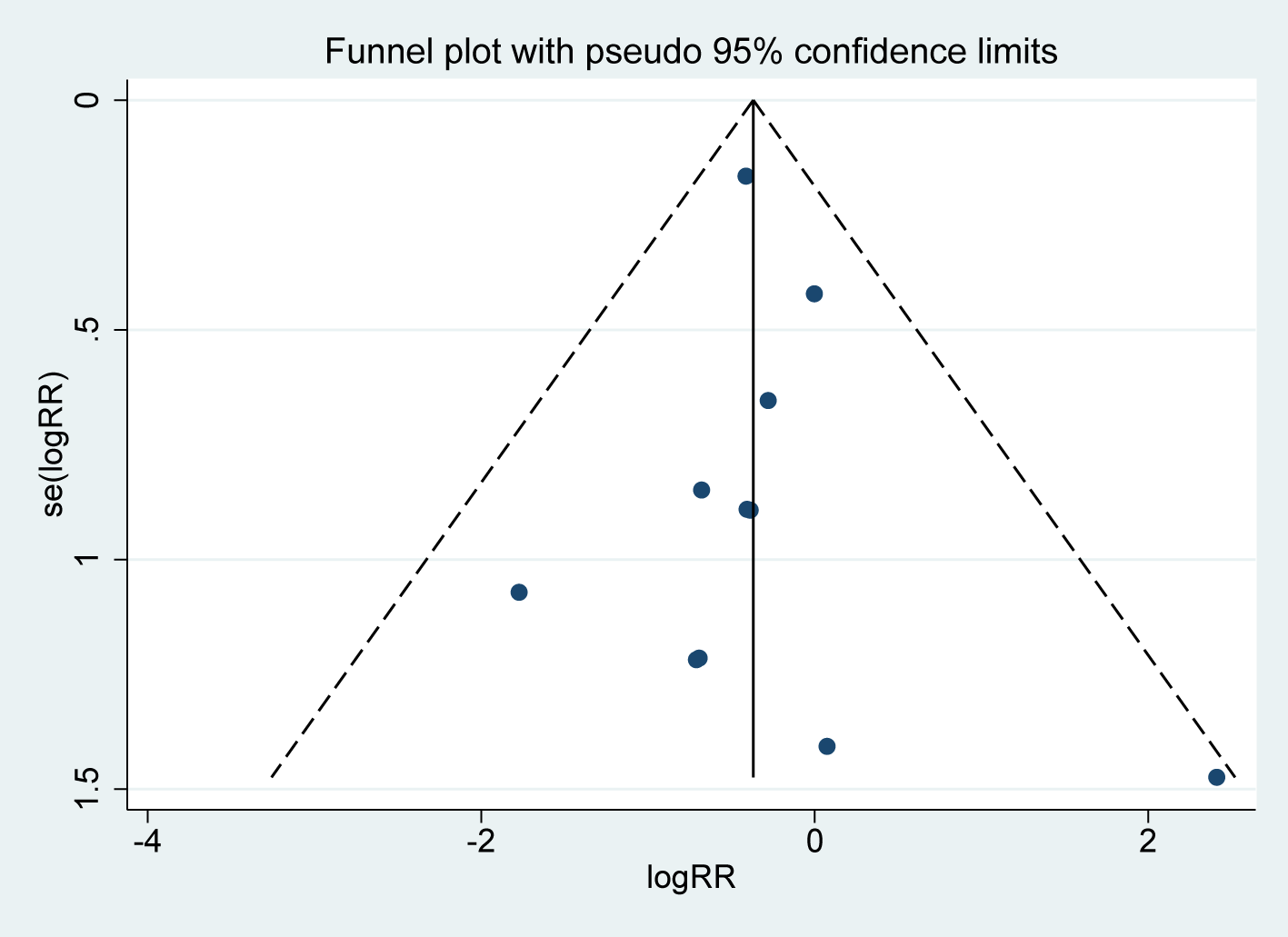

Supplement: Supplementary file 7 — Figure S3. A funnel plot of the risk of postoperative short-term mortality. (TIF 299 kb) [file 12902_2018_268_MOESM7_ESM.tif]

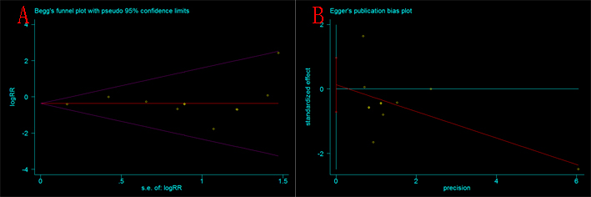

Supplement: Supplementary file 8 — Figure S4. A: Begg’s test for short-term mortality; B: Egger’s test for short-term mortality. (TIF 365 kb) [file 12902_2018_268_MOESM8_ESM.tif]

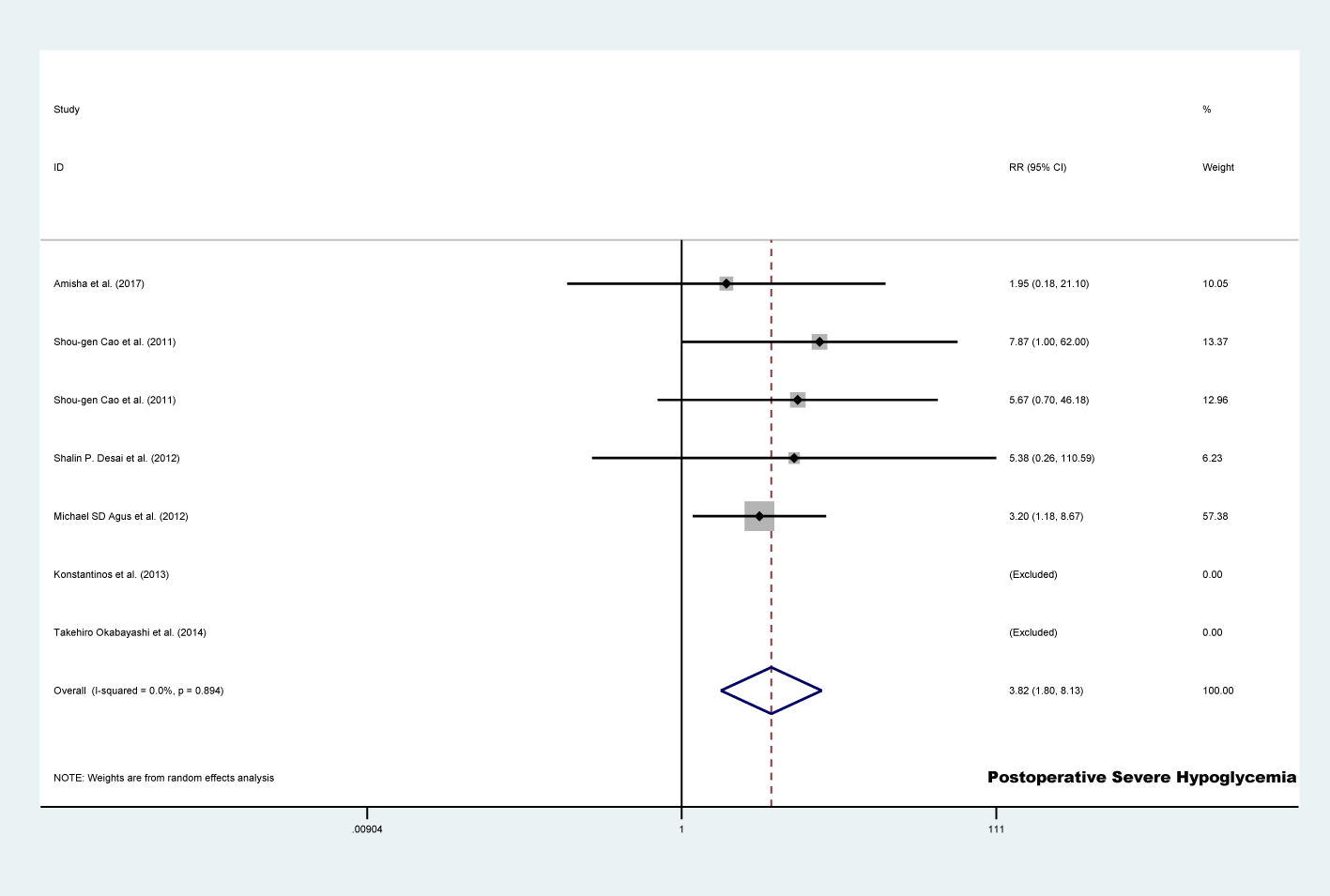

Supplement: Supplementary file 11 — Figure S5. Forest plot of the risk of postoperative servese hypoglycemia in TGC group versus control group. TGC = tight glycemic control; RR = relative risk; CI = confidence interval. (TIF 300 kb) [file 12902_2018_268_MOESM11_ESM.tif]

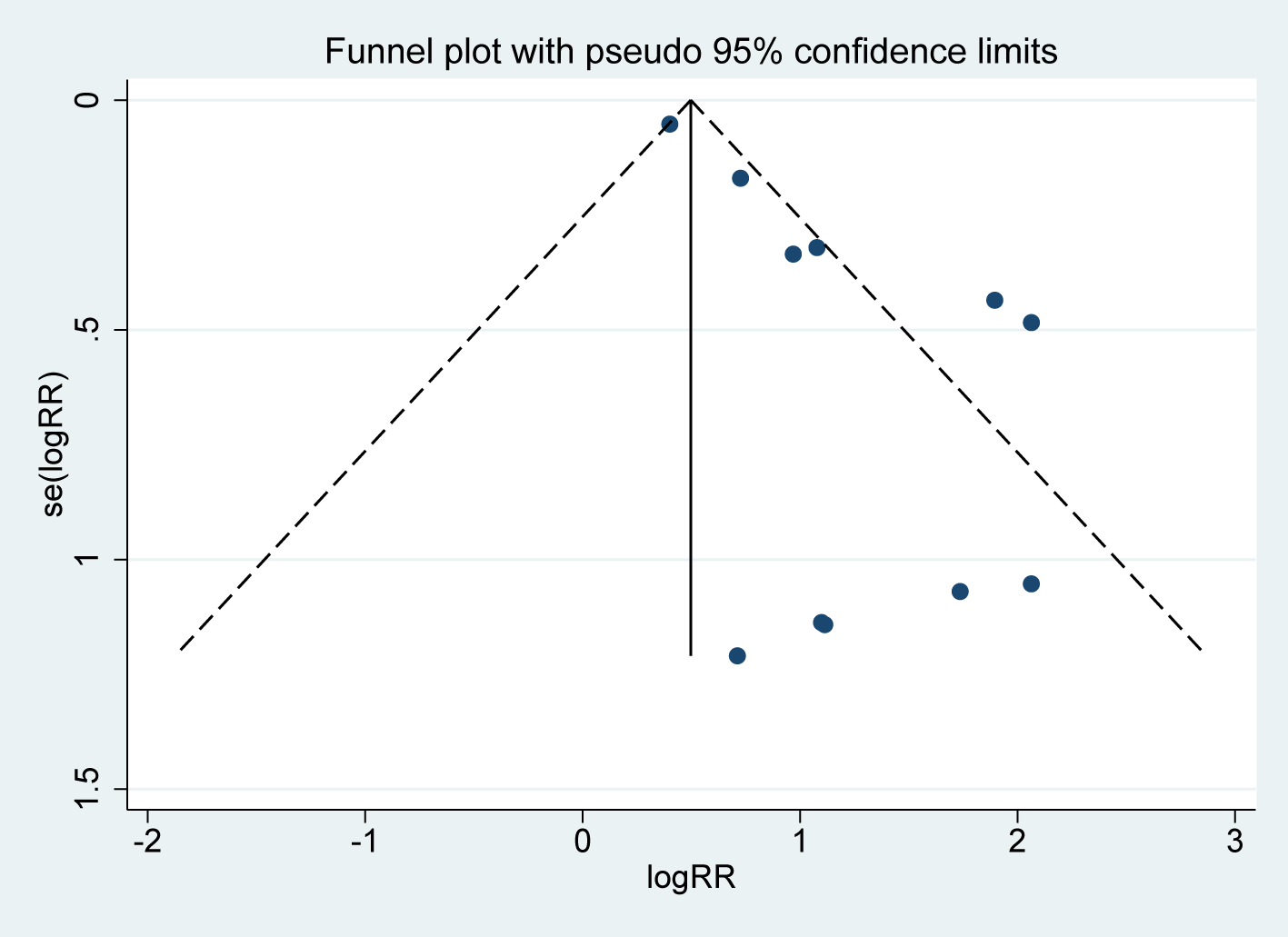

Supplement: Supplementary file 13 — Figure S6. A funnel plot of the risk of postoperative hypoglycemia. (TIF 293 kb) [file 12902_2018_268_MOESM13_ESM.tif]

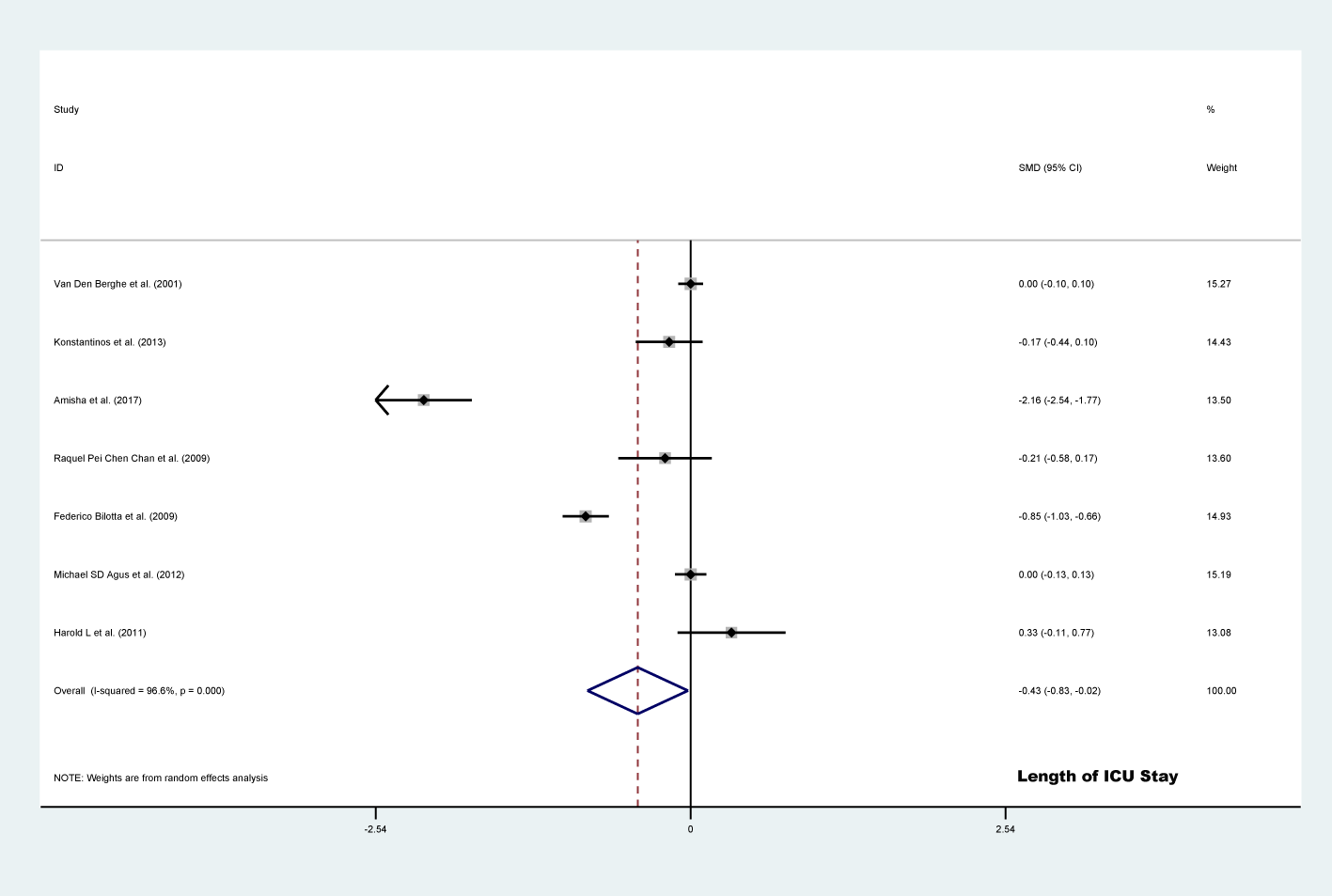

Supplement: Supplementary file 17 — Figure S7. Forest plot of the risk of postoperative ICU stay in TGC group versus control group. TGC = tight glycemic control; SMD = standardised mean difference; CI = confidence interval . (TIF 296 kb) [file 12902_2018_268_MOESM17_ESM.tif]

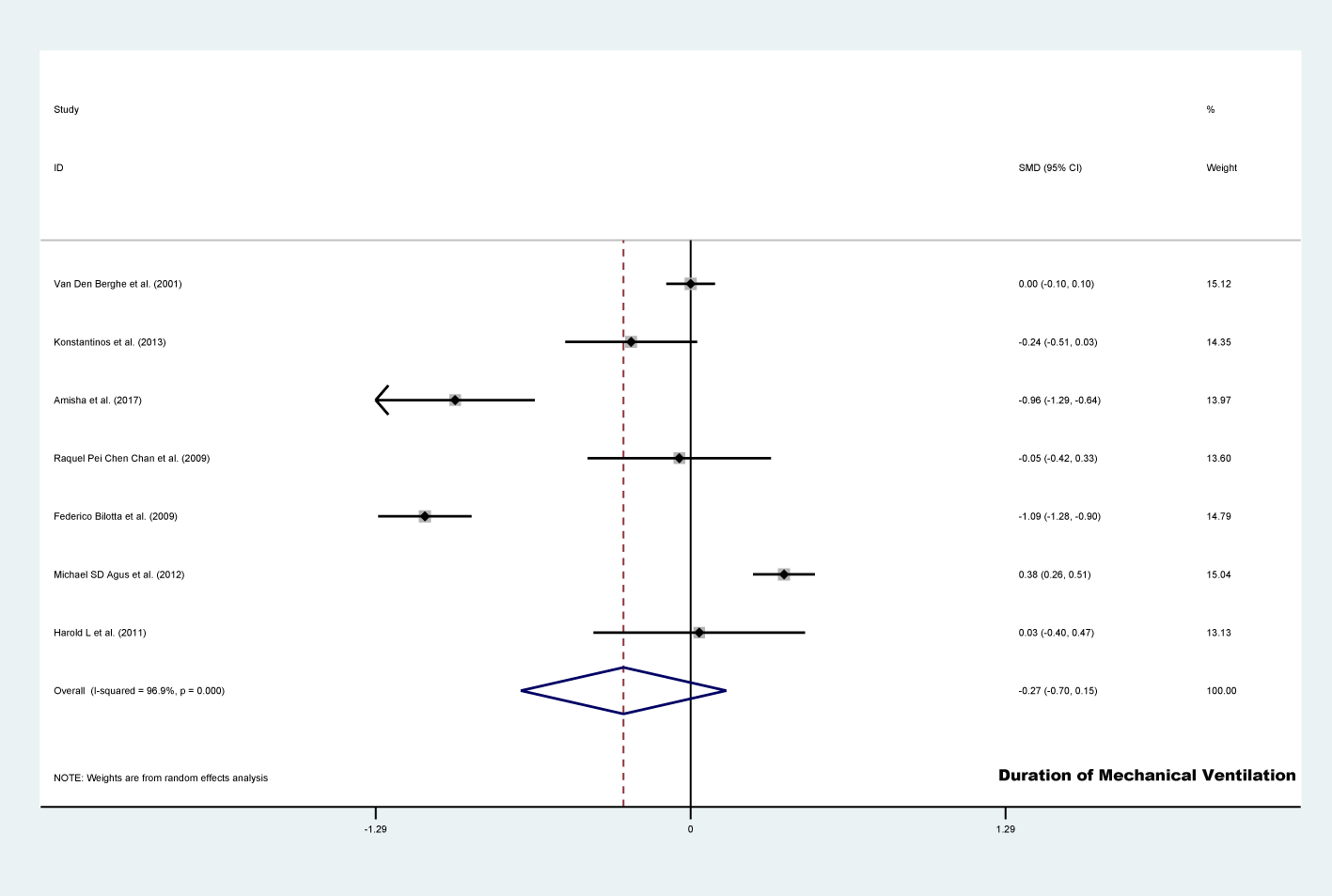

Supplement: Supplementary file 18 — Figure S8. Forest plot of the risk of postoperative duration of mechanical ventilation in TGC group versus control group. TGC = tight glycemic control; SMD = standardised mean difference; CI = confidence interval. (TIF 300 kb) [file 12902_2018_268_MOESM18_ESM.tif]

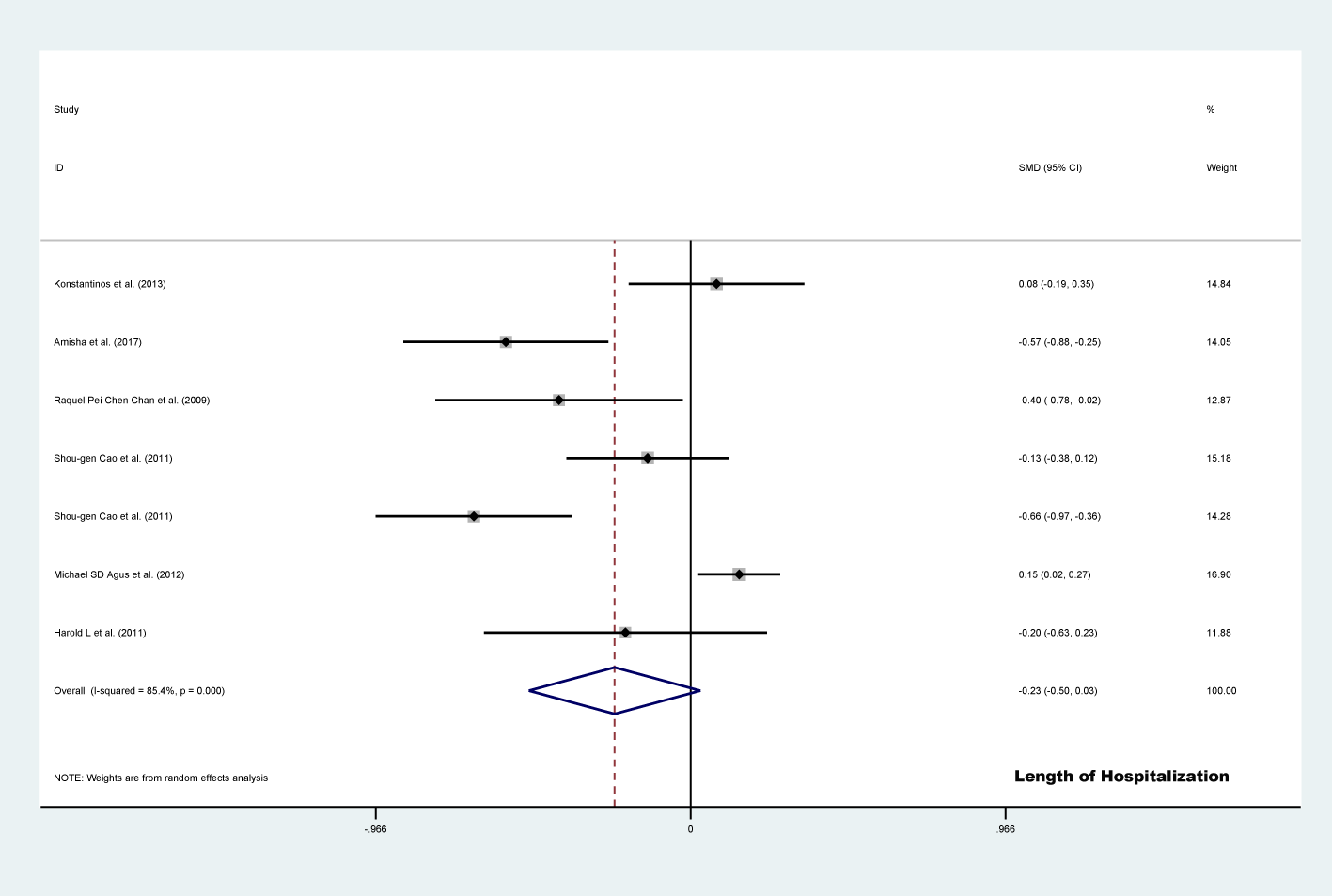

Supplement: Supplementary file 19 — Figure S9. Forest plot of the risk of postoperative LOS in TGC group versus control group. TGC = tight glycemic control; SMD = standardised mean difference; CI = confidence interval; LOS = length of hospitalization. (TIF 297 kb) [file 12902_2018_268_MOESM19_ESM.tif]
