# Supplementary material for: Postoperative tight glycemic control significantly reduces postoperative infection rates in patients undergoing surgery: a meta-analysis
Source: BMC Endocr Disord. 2018 Jun 22;18:42. doi: 10.1186/s12902-018-0268-9 (PMC6013895; doi:10.1186/s12902-018-0268-9)
Supplement: Supplementary file 6 — Table S2. Sensitivity analysis for the outcome of the risk of postoperative short-term mortality. (DOC 48 kb) [file 12902_2018_268_MOESM6_ESM.doc]

**Supplemental table 2. Sensitivity analysisfor the outcome of the risk of postoperative short-term mortality.**

| **Study omitted** | **Estimate RR** | **95% CI** | | ***P* value** | **Heterogeneity** | |
| --- | --- | --- | --- | --- | --- | --- |
|  |  | **Lower** | **Upper** |  | **I^2^ (%)** | ***P* value** |
| Van Den Berghe et al. (2001) | 0.769 | 0.464 | 1.275 | 0.309 | < 0.001 | 0.709 |
| Salah M et al. (2013) | 0.692 | 0.527 | 0.909 | 0.008 | < 0.001 | 0.769 |
| Konstantinos et al. (2013) | 0.709 | 0.538 | 0.933 | 0.014 | < 0.001 | 0.849 |
| Amisha et al. (2017) | 0.695 | 0.528 | 0.915 | 0.009 | < 0.001 | 0.693 |
| Rehong Zheng et al. (2010) | 0.693 | 0.525 | 0.913 | 0.009 | < 0.001 | 0.686 |
| Raquel Pei Chen Chan et al. (2009) | 0.692 | 0.525 | 0.912 | 0.009 | < 0.001 | 0.686 |
| Shou-gen Cao et al. (2011) | 0.695 | 0.528 | 0.915 | 0.009 | < 0.001 | 0.694 |
| Shou-gen Cao et al. (2011) | 0.689 | 0.521 | 0.911 | 0.009 | < 0.001 | 0.688 |
| Takehiro Okabayashi et al. (2014) | 0.675 | 0.513 | 0.888 | 0.005 | < 0.001 | 0.967 |
| Ehab A. Wahby et al. (2016) | 0.698 | 0.529 | 0.920 | 0.011 | < 0.001 | 0.700 |
| Shalin P. Desai et al. (2012) | 0.689 | 0.524 | 0.906 | 0.008 | < 0.001 | 0.696 |
| Michael SD Agus et al. (2012) | 0.662 | 0.495 | 0.883 | 0.005 | < 0.001 | 0.771 |
| Harold L et al. (2011) | 0.692 | 0.527 | 0.909 | 0.008 | < 0.001 | 0.769 |
| Combined | 0.692 | 0.527 | 0.909 | 0.008 | < 0.001 | 0.769 |

RR, Relative risk; CI, Confidence interval.
