# Supplementary material for: Postoperative tight glycemic control significantly reduces postoperative infection rates in patients undergoing surgery: a meta-analysis
Source: BMC Endocr Disord. 2018 Jun 22;18:42. doi: 10.1186/s12902-018-0268-9 (PMC6013895; doi:10.1186/s12902-018-0268-9)
Supplement: Supplementary file 9 — Table S3. Sensitivity analysis for the outcome of the risk of postoperative neurological dysfunction. (DOC 38 kb) [file 12902_2018_268_MOESM9_ESM.doc]

**Supplemental table 3. Sensitivity analysisfor the outcome of the risk of postoperative neurological dysfunction.**

| **Study omitted** | **Estimate RR** | **95% CI** | | ***P* value** | **Heterogeneity** |  |
| --- | --- | --- | --- | --- | --- | --- |
|  |  | **Lower** | **Upper** |  | **I^2^ (%)** | ***P* value** |
| Salah M et al. (2013) | 0.505 | 0.219 | 1.162 | 0.108 | < 0.001 | 0.537 |
| Raquel Pei Chen Chan et al. (2009) | 0.559 | 0.233 | 1.344 | 0.194 | < 0.001 | 0.669 |
| Ehab A. Wahby et al. (2016) | 0.335 | 0.116 | 0.966 | 0.043 | < 0.001 | 0.723 |
| Shalin P. Desai et al. (2012) | 0.464 | 0.209 | 1.030 | 0.059 | < 0.001 | 0.651 |
| Michael SD Agus et al. (2012) | 0.446 | 0.168 | 1.188 | 0.106 | < 0.001 | 0.437 |
| Combined | 0.499 | 0.219 | 1.137 | 0.098 | < 0.001 | 0.651 |

RR, Relative risk; CI, Confidence interval.
