# Supplementary material for: Postoperative tight glycemic control significantly reduces postoperative infection rates in patients undergoing surgery: a meta-analysis
Source: BMC Endocr Disord. 2018 Jun 22;18:42. doi: 10.1186/s12902-018-0268-9 (PMC6013895; doi:10.1186/s12902-018-0268-9)
Supplement: Supplementary file 10 — Table S4. Sensitivity analysis for the outcome of the risk of postoperative acute renal failure. (DOC 35 kb) [file 12902_2018_268_MOESM10_ESM.doc]

**Supplemental table 4. Sensitivity analysisfor the outcome of the risk of postoperative acute renal failure.**

| **Study omitted** | **Estimate RR** | **95% CI** | | ***P* value** | **Heterogeneity** |  |
| --- | --- | --- | --- | --- | --- | --- |
|  |  | **Lower** | **Upper** |  | **I^2^ (%)** | ***P* value** |
| Ehab A. Wahby et al. (2016) | 1.248 | 0.272 | 5.715 | 0.776 | 22.9 | 0.255 |
| Shalin P. Desai et al. (2012) | 0.507 | 0.160 | 1.608 | 0.249 | 32.9 | 0.222 |
| Michael SD Agus et al. (2012) | 0.877 | 0.045 | 16.981 | 0.931 | 68.6 | 0.074 |
| Combined | 0.703 | 0.191 | 2.589 | 0.596 | 43.5 | 0.171 |

RR, Relative risk; CI, Confidence interval.
