# Supplementary material for: Postoperative tight glycemic control significantly reduces postoperative infection rates in patients undergoing surgery: a meta-analysis
Source: BMC Endocr Disord. 2018 Jun 22;18:42. doi: 10.1186/s12902-018-0268-9 (PMC6013895; doi:10.1186/s12902-018-0268-9)
Supplement: Supplementary file 12 — Table S5. Sensitivity analysis for the outcome of the risk of postoperative hypoglycemia. (DOC 47 kb) [file 12902_2018_268_MOESM12_ESM.doc]

| **Study omitted** | **Estimate RR** | **95% CI** | | ***P* value** | **Heterogeneity** |  |
| --- | --- | --- | --- | --- | --- | --- |
|  |  | **Lower** | **Upper** |  | **I^2^ (%)** | ***P* value** |
| Van Den Berghe et al. (2001) | 2.749 | 1.750 | 4.321 | < 0.001 | 75.1 | < 0.001 |
| Amisha et al. (2017) | 3.257 | 1.896 | 5.594 | < 0.001 | 82.3 | < 0.001 |
| Rehong Zheng et al. (2010) | 3.158 | 1.910 | 5.222 | < 0.001 | 83.1 | < 0.001 |
| Raquel Pei et al. (2009) | 3.204 | 1.936 | 5.303 | < 0.001 | 83.3 | < 0.001 |
| Shou-gen Cao et al. (2011) | 3.008 | 1.843 | 4.912 | < 0.001 | 82.0 | < 0.001 |
| Shou-gen Cao et al. (2011) | 3.062 | 1.864 | 5.030 | < 0.001 | 82.5 | < 0.001 |
| Ehab A. Wahby et al. (2016) | 3.156 | 1.909 | 5.217 | < 0.001 | 83.1 | < 0.001 |
| Federico Bilotta et al. (2009) | 3.361 | 2.311 | 4.890 | < 0.001 | 33.9 | 0.137 |
| Shalin P. Desai et al. (2012) | 3.194 | 1.871 | 5.450 | < 0.001 | 81.5 | < 0.001 |
| Michael SD Agus et al. (2012) | 3.554 | 1.815 | 6.960 | < 0.001 | 83.0 | < 0.001 |
| Harold L et al. (2011) | 2.734 | 1.736 | 4.305 | < 0.001 | 75.9 | < 0.001 |
| Combined | 2.749 | 1.750 | 4.321 | < 0.001 | 75.1 | < 0.001 |

**Supplemental table 5. Sensitivity analysis for the outcome of the risk of any postoperative hypoglycemia**

RR, Relative risk; CI, Confidence interval.
