# Supplementary material for: Postoperative tight glycemic control significantly reduces postoperative infection rates in patients undergoing surgery: a meta-analysis
Source: BMC Endocr Disord. 2018 Jun 22;18:42. doi: 10.1186/s12902-018-0268-9 (PMC6013895; doi:10.1186/s12902-018-0268-9)
Supplement: Supplementary file 14 — Table S6. Sensitivity analysis for the outcome of the risk of postoperative servese hypoglycemia. (DOC 40 kb) [file 12902_2018_268_MOESM14_ESM.doc]

**Supplemental table 6. Sensitivity analysisfor the outcome of the risk of severe postoperative hypoglycemia**

| **Study omitted** | **Estimate RR** | **95% CI** | | ***p-*value** | **Heterogeneity** |  |
| --- | --- | --- | --- | --- | --- | --- |
|  |  | **Lower** | **Upper** |  | **I^2^ (%)** | ***P* value** |
| Konstantinos et al. (2013) | 3.821 | 1.796 | 8.127 | < 0.001 | < 0.001 | 0.894 |
| Amisha et al. (2017) | 4.119 | 1.858 | 9.128 | < 0.001 | < 0.001 | 0.860 |
| Shou-gen Cao et al. (2011) | 3.417 | 1.519 | 7.689 | 0.003 | < 0.001 | 0.909 |
| Shou-gen Cao et al. (2011) | 3.602 | 1.604 | 8.089 | 0.002 | < 0.001 | 0.815 |
| Takehiro Okabayashi et al. (2014) | 3.821 | 1.796 | 8.127 | < 0.001 | < 0.001 | 0.894 |
| Shalin P. Desai et al. (2012) | 3.735 | 1.713 | 8.142 | 0.001 | < 0.001 | 0.789 |
| Michael SD Agus et al. (2012) | 4.851 | 1.526 | 15.414 | 0.007 | < 0.001 | 0.846 |
| Combined | 3.821 | 1.796 | 8.127 | < 0.001 | < 0.001 | 0.894 |

RR, Relative risk; CI, Confidence interval.
