# Supplementary material for: Postoperative tight glycemic control significantly reduces postoperative infection rates in patients undergoing surgery: a meta-analysis
Source: BMC Endocr Disord. 2018 Jun 22;18:42. doi: 10.1186/s12902-018-0268-9 (PMC6013895; doi:10.1186/s12902-018-0268-9)
Supplement: Supplementary file 15 — Table S7. Subgroup analyses for the outcome of the risk of postoperative hypoglycemia. (DOC 68 kb) [file 12902_2018_268_MOESM15_ESM.doc]

**Supplemental table 7. Subgroup analysisfor the outcome of the risk of post-operative hypoglycemia.**

| **Group** | **Studies** | **TGC** |  | **CGC** |  | **M-H pooled RR** |  |  | **Heterogeneity** | |
| --- | --- | --- | --- | --- | --- | --- | --- | --- | --- | --- |
|  |  | **N+**(%) | **Total** | **N+**(%) | **Total** | **RR (95%CI)** | **z** | **p** | **I^2^ (%)** | **p** |
| Total | 11 | 467(32.9) | 2097 | 233(11.0) | 2118 | 3.145 (1.928, 5.131) | 4.59 | <0.001 | 81.4 | <0.001 |
| **Type of Surgery** |  |  |  |  |  |  |  |  |  |  |
| Various surgeries | 1 | 39(5.1) | 765 | 6(0.8) | 783 | 6.653 (2.833, 15.623) | 4.35 | <0.001 | NA | NA |
| Liver transplantation | 1 | 27(32.9) | 82 | 10(12.5) | 80 | 2.634 (1.366, 5.081) | 2.89 | 0.004 | NA | NA |
| Neurosurgery | 1 | 226(93.8) | 241 | 152(62.8) | 242 | 1.493 (1.348, 1.654) | 7.68 | <0.001 | NA | NA |
| Cardiac surgery | 6 | 161(20.3) | 792 | 63(7.8) | 803 | 2.924 (1.855, 4.607) | 4.62 | <0.001 | 31.4 | 0.200 |
| Radical gastrectomy | 2 | 14(6.5) | 217 | 2(0.95) | 210 | 6.700 (1.539, 29.166) | 2.53 | 0.011 | <0.001 | 0.827 |
| **Neurosurgery** |  |  |  |  |  |  |  |  |  |  |
| Yes | 1 | 226(93.8) | 241 | 152(62.8) | 242 | 1.493 (1.348, 1.654) | 7.68 | <0.001 | NA | NA |
| No | 10 | 241(13.0) | 1856 | 81(4.3) | 1876 | 3.361 (2.311, 4.890) | 6.34 | <0.001 | 33.9 | 0.137 |
| **Preoperative diabetes** | | | | | | | | | | |
| Yes | 7 | 175(11.5) | 1524 | 58(3.1) | 1876 | 3.511 (1.661,7.421) | 3.29 | 0.001 | 87.4 | <0.001 |
| No | 4 | 292(51.0) | 573 | 175(30.4) | 575 | 2.151 (1.559, 2.970) | 4.66 | <0.001 | <0.001 | 0.640 |
| **Type of patient** |  |  |  |  |  |  |  |  |  |  |
| Adult | 10 | 374(23.3) | 1607 | 188(10.0) | 1876 | 3.554 (1.815, 6.960) | 3.70 | <0.001 | 83.0 | <0.001 |
| Birth to 36 months | 1 | 93(19.0) | 490 | 45(9.2) | 490 | 2.067 (1.481, 2.884) | 4.27 | <0.001 | NA | NA |
| **Time of intervention** |  |  |  |  |  |  |  |  |  |  |
| Postoperative | 7 | 429(22.7) | 1886 | 226(11.9) | 1903 | 2.770 (1.685, 4.554) | 4.02 | <0.001 | 83.5 | <0.001 |
| Intra + Post operative | 4 | 38(18.0) | 211 | 7(3.3) | 215 | 5.441 (2.520,11.744) | 4.31 | <0.001 | <0.001 | 0.619 |
| **Trigger of blood glucose(mg/dL)** | | | | | | | | | | |
| ≤110 | 5 | 375(21.3) | 1763 | 206(11.6) | 1775 | 2.795 (1.534, 5.095) | 3.36 | 0.001 | 81.8 | <0.001 |
| 110-150 | 3 | 62(33.5) | 185 | 16(8.2) | 195 | 4.081 (1.901,8.763) | 3.61 | <0.001 | 38.3 | 0.198 |
| ≥150 | 2 | 30(20.1) | 149 | 11(7.4) | 148 | 2.665 (1.419,5.005) | 3.05 | 0.002 | <0.001 | 0.903 |
| **Use of glucocorticoids in hospital** | | | | | | | | | | |
| Yes | 3 | 346(42.6) | 813 | 207(25.5) | 812 | 1.866 (1.280, 2.720) | 3.25 | 0.001 | 75.5 | 0.017 |
| No | 8 | 82(15.8) | 519 | 20(3.8) | 523 | 4.473 (2.978,6.717) | 7.22 | <0.001 | <0.001 | 0.654 |
| **Jadad score** |  |  |  |  |  |  |  |  |  |  |
| low quality:1-3 points | 7 | 82(15.8) | 519 | 20(3.8) | 523 | 3.981 (2.507,6.321) | 5.86 | <0.001 | <0.001 | 0.686 |
| high quality: 4-7 points | 4 | 346(42.6) | 813 | 207(25.5) | 812 | 2.426 (1.405, 4.189) | 3.18 | 0.001 | 87.8 | <0.001 |

N+: The number of exposure to TGC; Total, The number of the total patients; RR, Relative risk; NR, not reported.
