# Supplementary material for: Postoperative tight glycemic control significantly reduces postoperative infection rates in patients undergoing surgery: a meta-analysis
Source: BMC Endocr Disord. 2018 Jun 22;18:42. doi: 10.1186/s12902-018-0268-9 (PMC6013895; doi:10.1186/s12902-018-0268-9)
Supplement: Supplementary file 16 — Table S8. Meta-regression for the outcome of the risk of postoperative hypoglycemia. (DOC 44 kb) [file 12902_2018_268_MOESM16_ESM.doc]

**Supplemental table 8. Meta-regression for the outcome of the risk of postoperative hypoglycemia.**

| **Sources** | **Coefficient (95%CI)** | **t** | ***p*** | **τ^2^** | **I^2^ Res (%)** | **Adjusted R^2^ (%)** |
| --- | --- | --- | --- | --- | --- | --- |
| Type of surgery | - 0.23 (-0.58, 0.12) | -1.51 | 0.165 | 0.162 | 39.97 | 33.66 |
| Neurosurgical | -0.84 (-1.84, 0.16) | -1.90 | 0.090 | 0.148 | 31.98 | 39.11 |
| Type of patient | -0.48 (-1.87, 0.92) | -0.78 | 0.458 | 0.287 | 73.07 | -17.80 |
| Time of intervention | 0.61 (-0.58, 1.80) | 1.15 | 0.279 | 0.183 | 65.32 | 24.99 |
| Preoperative diabetes | 0.23 (-0.94, 1.39) | 0.44 | 0.673 | 0.282 | 72.28 | -15.95 |
| Trigger of blood glucose | 0.06 (-0.62, 0.75) | 0.21 | 0.840 | 0.280 | 65.40 | -15.14 |
| Use of glucocorticoids in hospital | -0.92 (-1.57, -0.27) | -3.21 | 0.011 | 0.051 | 16.59 | 78.96 |
| Age | 0.01 (-0.01, 0.03) | 1.00 | 0.343 | 0.271 | 73.83 | -11.50 |
| Sample size | -0.0008 (-0.0010, 0.0012) | 0.16 | 0.873 | 0.289 | 73.44 | -18.88 |
| Jadad Score | -0.14 (-0.34, 0.07) | -1.51 | 0.166 | 0.174 | 51.05 | 28.67 |

CI, Confidence interval.
