# Supplementary material for: Postoperative tight glycemic control significantly reduces postoperative infection rates in patients undergoing surgery: a meta-analysis
Source: BMC Endocr Disord. 2018 Jun 22;18:42. doi: 10.1186/s12902-018-0268-9 (PMC6013895; doi:10.1186/s12902-018-0268-9)
Supplement: Supplementary file 20 — Table S9. Sensitivity analysis for the outcome of the risk of postoperative ICU stay. (DOC 40 kb) [file 12902_2018_268_MOESM20_ESM.doc]

**Supplemental table 9. Sensitivity analysisfor the outcome of postoperative ICU stay.**

| **Study omitted** | **Estimate SMD** | **95% CI** | | ***P* value** | **Heterogeneity** |  |
| --- | --- | --- | --- | --- | --- | --- |
|  |  | **Lower** | **Upper** |  | **I^2^ (%)** | ***P* value** |
| Van Den Berghe et al. (2001) | -0.506 | -1.062 | -0.049 | 0.074 | 96.7 | < 0.001 |
| Konstantinos et al. (2013) | -0.471 | -0.935 | -0.008 | 0.046 | 97.1 | < 0.001 |
| Amisha et al. (2017) | -0.166 | -0.460 | 0.128 | 0.269 | 93.1 | < 0.001 |
| Raquel Pei Chen Chan et al. (2009) | -0.463 | -0.913 | -0.013 | 0.044 | 97.1 | < 0.001 |
| Federico Bilotta et al. (2009) | -0.352 | -0.762 | 0.058 | 0.093 | 95.8 | < 0.001 |
| Michael SD Agus et al. (2012) | -0.506 | -1.055 | 0.043 | 0.071 | 96.9 | < 0.001 |
| Harold L et al. (2011) | -0.542 | -0.980 | -0.103 | 0.015 | 97.0 | < 0.001 |
| Combined | -0.428 | -0.833 | -0.022 | 0.039 | 96.6 | < 0.001 |

SMD, standardised mean difference; CI, Confidence interval.
