# Supplementary material for: Postoperative tight glycemic control significantly reduces postoperative infection rates in patients undergoing surgery: a meta-analysis
Source: BMC Endocr Disord. 2018 Jun 22;18:42. doi: 10.1186/s12902-018-0268-9 (PMC6013895; doi:10.1186/s12902-018-0268-9)
Supplement: Supplementary file 21 — Table S10. Sensitivity analysis for the outcome of the risk of postoperative duration of mechanical ventilation. (DOC 40 kb) [file 12902_2018_268_MOESM21_ESM.doc]

**Supplemental table 10. Sensitivity analysisfor the outcome of postoperative duration of tracheal intubation.**

| **Study omitted** | **Estimate SMD** | **95% CI** | | ***P* value** | **Heterogeneity** |  |
| --- | --- | --- | --- | --- | --- | --- |
|  |  | **Lower** | **Upper** |  | **I^2^ (%)** | ***P* value** |
| Van Den Berghe et al. (2001) | -0.323 | -0.930 | 0.284 | 0.298 | 97.3 | < 0.001 |
| Konstantinos et al. (2013) | -0.280 | -0.759 | 0.199 | 0.252 | 97.4 | < 0.001 |
| Amisha et al. (2017) | -0.163 | -0.598 | 0.272 | 0.463 | 96.9 | < 0.001 |
| Raquel Pei Chen Chan et al. (2009) | -0.311 | -0.777 | 0.156 | 0.191 | 97.4 | < 0.001 |
| Federico Bilotta et al. (2009) | -0.126 | -0.440 | 0.188 | 0.432 | 92.8 | < 0.001 |
| Michael SD Agus et al. (2012) | -0.391 | -0.855 | 0.073 | 0.099 | 95.8 | < 0.001 |
| Harold L et al. (2011) | -0.322 | -0.784 | 0.140 | 0.172 | 97.4 | < 0.001 |
| Combined | -0.275 | -0.695 | 0.146 | 0.201 | 96.9 | < 0.001 |

SMD, standardised mean difference; CI, Confidence interval.
