# Supplementary material for: Postoperative tight glycemic control significantly reduces postoperative infection rates in patients undergoing surgery: a meta-analysis
Source: BMC Endocr Disord. 2018 Jun 22;18:42. doi: 10.1186/s12902-018-0268-9 (PMC6013895; doi:10.1186/s12902-018-0268-9)
Supplement: Supplementary file 22 — Table S11. Sensitivity analysis for the outcome of the risk of postoperative length of hospitalization. (DOC 41 kb) [file 12902_2018_268_MOESM22_ESM.doc]

**Supplemental table 11. Sensitivity analysisfor the outcome of postoperative LOS stay.**

| **Study omitted** | **Estimate SMD** | **95% CI** | | ***P* value** | **Heterogeneity** |  |
| --- | --- | --- | --- | --- | --- | --- |
|  |  | **Lower** | **Upper** |  | **I^2^ (%)** | ***P* value** |
| Konstantinos et al. (2013) | -0.290 | -0.600 | 0.01 | 0.066 | 87.4 | < 0.001 |
| Amisha et al. (2017) | -0.176 | -0.441 | 0.089 | 0.192 | 83.6 | < 0.001 |
| Raquel Pei Chen Chan et al. (2009) | -0.209 | -0.495 | 0.077 | 0.153 | 86.8 | < 0.001 |
| Shou-gen Cao et al. (2011) | -0.255 | -0.573 | 0.063 | 0.116 | 87.7 | < 0.001 |
| Shou-gen Cao et al. (2011) | -0.154 | -0.394 | 0.086 | 0.209 | 79.5 | < 0.001 |
| Michael SD Agus et al. (2012) | -0.309 | -0.555 | -0.064 | 0.013 | 72.5 | 0.003 |
| Harold L et al. (2011) | -0.239 | -0.531 | 0.052 | 0.108 | 87.7 | < 0.001 |
| Combined | -0.233 | -0.496 | 0.030 | 0.082 | 85.4 | < 0.001 |

LOS, length of hospital stay; SMD, standardised mean difference; CI, Confidence interval.
