# Supplementary material for: Postoperative tight glycemic control significantly reduces postoperative infection rates in patients undergoing surgery: a meta-analysis
Source: BMC Endocr Disord. 2018 Jun 22;18:42. doi: 10.1186/s12902-018-0268-9 (PMC6013895; doi:10.1186/s12902-018-0268-9)
Supplement: Supplementary file 23 — Table S12. Subgroup analyses for the outcome of the risk of postoperative ICU stay. (DOC 55 kb) [file 12902_2018_268_MOESM23_ESM.doc]

**Supplemental table 12. Subgroup analysisfor the outcome of the risk of postoperative ICU stay.**

| **Group** | **Number of**  **studies** | **TGC** |  | **CGC** |  | **M-H pooled SMD** |  | **Heterogeneity** |  |
| --- | --- | --- | --- | --- | --- | --- | --- | --- | --- |
|  |  | **Mean ±SD** | **Total** | **Mean ±SD** | **Total** | **SMD (95%CI)** | ***p*** | **I^2^ (%)** | ***p*** |
| Total | 7 | 5±9 | 1777 | 6±13 | 1798 | -0.43 (-0.83, -0.02) | 0.039 | 96.6 | <0.001 |
| **Type of Surgery** |  |  |  |  |  |  |  |  |  |
| Various surgeries | 1 | 3±3 | 765 | 3±5 | 783 | <0.01 (-0.10, 0.10) | 1.000 | NR | NR |
| Liver transplantation | 1 | 47±6 | 82 | 59±6 | 80 | -2.16 (-2.54, -1.77) | <0.001 | NR | NR |
| Neurosurgery | 1 | 6±2 | 241 | 8±3 | 242 | -0.85 (-1.03, -0.66) | <0.001 | NR | NR |
| Cardiac surgery | 4 | 3±3 | 689 | 3±4 | 693 | 0.05 (-0.16, 0.07) | 0.692 | 36.9 | 0.191 |
| **Type of patient** |  |  |  |  |  |  |  |  |  |
| Adult | 6 | 6±11 | 1287 | 7±14 | 1309 | -0.51 (-1.06, 0.04) | 0.071 | 96.9 | <0.001 |
| Birth to 36 months | 1 | 3±3 | 490 | 3±3 | 489 | <0.001(-0.13, 0.13) | 1.000 | NR | NR |
| **Time of intervention** |  |  |  |  |  |  |  |  |  |
| Postoperative | 5 | 6±10 | 1683 | 6±13 | 1701 | -0.61 (-1.10, -0.11) | 0.016 | 97.6 | <0.001 |
| Intra + Post operative | 2 | 4±5 | 94 | 5±8 | 97 | 0.05 (-0.48,0.58) | 0.852 | 70.0 | 0.068 |
| **Trigger of blood glucose(mg/dL)** | | | | | | | | | |
| ≤110 | 3 | 3±3 | 1496 | 4±6 | 1514 | -0.28 (-0.72, 0.17) | 0.223 | 97.0 | <0.001 |
| 110-150 | 2 | 4±5 | 94 | 5±8 | 97 | 0.05 (-0.48,0.58) | 0.852 | 70.0 | 0.068 |
| ≥150 | 2 | 22±22 | 187 | 27±28 | 187 | -1.16 (-3.10,0.78) | 0.242 | 98.5 | 0.001 |
| **Preoperative diabetes** | | | | | | | | | |
| Yes | 5 | 6±12 | 1233 | 8±9 | 1254 | -0.57 (-1.20, 0.07) | 0.081 | 97.6 | <0.001 |
| No | 2 | 3±4 | 544 | 3±4 | 544 | -0.02 (-0.15,0.11) | 0.719 | 3.7 | 0.308 |
| **Use of glucocorticoids in hospital** | | | | | | | | | |
| Yes | 3 | 8±13 | 813 | 10±17 | 811 | -0.98 (-1.96, -0.01) | 0.048 | 98.6 | <0.001 |
| No | 4 | 3±3 | 964 | 3±5 | 987 | -0.03 (-0.19,0.13) | 0.697 | 37.6 | 0.187 |

Total, The number of the total patients; SMD, standardised mean difference; NR, not reported.
