# Supplementary material for: Postoperative tight glycemic control significantly reduces postoperative infection rates in patients undergoing surgery: a meta-analysis
Source: BMC Endocr Disord. 2018 Jun 22;18:42. doi: 10.1186/s12902-018-0268-9 (PMC6013895; doi:10.1186/s12902-018-0268-9)
Supplement: Supplementary file 24 — Table S13. Meta-regression for the outcome of the risk of postoperative ICU stay. (DOC 45 kb) [file 12902_2018_268_MOESM24_ESM.doc]

**Supplemental table 13. Meta-regression for the outcome of the risk of postoperative ICU stay.**

| **Sources** | **Coefficient (95%CI)** | **t** | ***P*** | **τ^2^** | **I^2^ Res (%)** | **Adjusted R^2^ (%)** |
| --- | --- | --- | --- | --- | --- | --- |
| Type of surgery | - 0.38 (-1.09, 0.34) | -1.36 | 0.233 | 0.569 | 94.45 | 13.06 |
| Type of patient | 0.51 (-1.93, 2.94) | 0.54 | 0.615 | 0.751 | 96.94 | -14.78 |
| Time of intervention | 0.68 (-1.13, 2.49) | 0.97 | 0.378 | 0.666 | 97.10 | -1.79 |
| Trigger of blood glucose | -0.39 (-1.35, 0.57) | -1.03 | 0.348 | 0.650 | 96.63 | 0.67 |
| Preoperative diabetes | -0.47 (-2.35, 1.42) | -0.64 | 0.553 | 0.731 | 96.96 | -11.76 |
| Use of glucocorticoids in hospital | -0.96 (-2.35, 0.43) | -1.77 | 0.137 | 0.481 | 96.55 | 26.49 |
| Jadad Score | -0.07 (-0.49, 0.34) | -0.46 | 0.664 | 0.763 | 97.12 | -16.70 |
| Year of publication | -0.09 (-0.25, 0.07) | -1.41 | 0.217 | 0.561 | 96.64 | 14.14 |
| Sample size | -0.0004 (-0.001, 0.002) | 0.65 | 0.542 | 0.727 | 95.97 | -11.12 |
| Age | 0.005 (-0.045, 0.036) | -0.30 | 0.779 | 0.782 | 97.04 | -19.60 |

CI, Confidence interval.
