# Supplementary material for: Postoperative tight glycemic control significantly reduces postoperative infection rates in patients undergoing surgery: a meta-analysis
Source: BMC Endocr Disord. 2018 Jun 22;18:42. doi: 10.1186/s12902-018-0268-9 (PMC6013895; doi:10.1186/s12902-018-0268-9)
Supplement: Supplementary file 25 — Table S14. Subgroup analyses for the outcome of the risk of postoperative duration of mechanical ventilation. (DOC 55 kb) [file 12902_2018_268_MOESM25_ESM.doc]

**Supplemental table 14. Subgroup analysis for the outcome of the risk of postoperative duration of tracheal intubation.**

| **Group** | **Number of**  **studies** | **TGC** |  | **CGC** |  | **M-H pooled SMD** |  | **Heterogeneity** |  |
| --- | --- | --- | --- | --- | --- | --- | --- | --- | --- |
|  |  | **Mean ±SD** | **Total** | **Mean ±SD** | **Total** | **SMD (95%CI)** | **p** | **I^2^ (%)** | **p** |
| Total | 7 | 57±59 | 1777 | 56±82 | 1798 | -0.28 (-0.70, 0.15) | 0.201 | 96.9 | <0.001 |
| **Type of Surgery** |  |  |  |  |  |  |  |  |  |
| Various surgeries | 1 | 48±53 | 765 | 48±89 | 783 | <0.01 (-0.10, 0.10) | 1.000 | NR | NR |
| Liver transplantation | 1 | 10±4 | 82 | 15±5 | 80 | -0.96 (-1.29, -0.64) | <0.001 | NR | NR |
| Neurosurgery | 1 | 101±34 | 241 | 146±49 | 242 | -1.09 (-1.28, -0.90) | <0.001 | NR | NR |
| Cardiac surgery | 4 | 56±65 | 689 | 40±67 | 693 | 0.05 (-0.31, 0.40) | 0.799 | 85.2 | <0.001 |
| **Type of patient** |  |  |  |  |  |  |  |  |  |
| Adult | 6 | 50±59 | 1287 | 60±86 | 1309 | -0.39 (-0.86, 0.07) | 0.099 | 95.8 | <0.001 |
| Birth to 36 months | 1 | 72±53 | 490 | 48±71 | 489 | 0.38 (0.26, 0.51) | <0.001 | NR | NR |
| **Time of intervention** | | | | | | | | | |
| Postoperative | 5 | 58±54 | 1683 | 59±83 | 1701 | -0.37 (-0.89, 0.15) | 0.159 | 97.9 | <0.001 |
| Intra + Post operative | 2 | 20±108 | 94 | 22±62 | 97 | -0.01 (-0.30,0.27) | 0.936 | <0.001 | 0.780 |
| **Trigger of blood glucose(mg/dL)** | | | | | | | | | |
| ≤110 | 3 | 64±54 | 1496 | 64±86 | 1514 | -0.23 (-0.92, 0.46) | 0.509 | 98.7 | <0.001 |
| 110-150 | 2 | 20±108 | 94 | 22±62 | 97 | -0.01 (-0.30,0.27) | 0.936 | <0.001 | 0.780 |
| ≥150 | 2 | 8±35 | 187 | 17±27 | 187 | -0.60 (-1.30,0.11) | 0.097 | 91.0 | 0.001 |
| **Preoperative diabetes** | | | | | | | | | |
| Yes | 5 | 51±52 | 1233 | 61±86 | 1254 | -0.46 (-0.99, 0.08) | 0.093 | 96.6 | <0.001 |
| No | 2 | 68±68 | 544 | 47±72 | 544 | 0.21 (-0.21,0.62) | 0.329 | 77.8 | 0.034 |
| **Use of glucocorticoids in hospital** | | | | | | | | | |
| Yes | 3 | 74±52 | 813 | 74±78 | 811 | -0.55 (-1.65, 0.54) | 0.323 | 98.9 | <0.001 |
| No | 4 | 41±59 | 964 | 42±83 | 987 | -0.03 (-0.12,0.06) | 0.545 | <0.001 | 0.418 |

Total, The number of the total patients; SMD, standardised mean difference; NA, not reported.
