# Supplementary material for: Postoperative tight glycemic control significantly reduces postoperative infection rates in patients undergoing surgery: a meta-analysis
Source: BMC Endocr Disord. 2018 Jun 22;18:42. doi: 10.1186/s12902-018-0268-9 (PMC6013895; doi:10.1186/s12902-018-0268-9)
Supplement: Supplementary file 26 — Table S15. Meta-regression for the outcome of the risk of postoperative duration of mechanical ventilation. (DOC 44 kb) [file 12902_2018_268_MOESM26_ESM.doc]

**Supplemental table 15. Meta-regression for the outcome of the risk of post-operative duration of tracheal intubation.**

| **Sources** | **Coefficient (95%CI)** | **t** | ***P*** | **τ^2^** | **I^2^ Res (%)** | **Adjusted R^2^ (%)** |
| --- | --- | --- | --- | --- | --- | --- |
| Type of surgery | - 0.40 (-0.70, -0.10) | -3.47 | 0.018 | 0.092 | 88.98 | 68.12 |
| Type of patient | 0.77 (-0.61, 2.16) | 1.44 | 0.209 | 0.241 | 95.81 | 16.88 |
| Time of intervention | 0.37 (-0.90, 1.64) | 0.74 | 0.491 | 0.315 | 97.37 | -8.63 |
| Trigger of blood glucose | -0.16 (-0.84, 0.52) | -0.60 | 0.575 | 0.326 | 97.11 | -12.61 |
| Preoperative diabetes | -0.64 (-1.72, 0.44) | -1.53 | 0.187 | 0.231 | 95.92 | 20.35 |
| Use of glucocorticoids in hospital | -0.48 (-1.55, 0.59) | -1.15 | 0.304 | 0.279 | 97.33 | 3.77 |
| Jadad Score | -0.03 (-0.31, 0.25) | -0.27 | 0.800 | 0.345 | 97.32 | -19.31 |
| Year of publication | 0.03 (-0.16, 0.09) | -0.69 | 0.519 | 0.321 | 97.36 | -10.75 |
| Sample size | -0.0004 (-0.008, 0.001) | 0.85 | 0.434 | 0.301 | 96.64 | -4.00 |
| Age | 0.01 (-0.03, 0.01) | -1.12 | 0.313 | 0.275 | 96.26 | 5.16 |

CI, Confidence interval.
