# Supplementary material for: Postoperative tight glycemic control significantly reduces postoperative infection rates in patients undergoing surgery: a meta-analysis
Source: BMC Endocr Disord. 2018 Jun 22;18:42. doi: 10.1186/s12902-018-0268-9 (PMC6013895; doi:10.1186/s12902-018-0268-9)
Supplement: Supplementary file 27 — Table S16. Subgroup analyses for the outcome of the risk of postoperative length of hospitalization. (DOC 60 kb) [file 12902_2018_268_MOESM27_ESM.doc]

**Supplemental table 16. Subgroup analysisfor the outcome of the risk of postoperative LOS.**

| **Group** | **Number of**  **studies** | **TGC** |  | **CGC** |  | **M-H pooled SMD** |  | **Heterogeneity** |  |
| --- | --- | --- | --- | --- | --- | --- | --- | --- | --- |
|  |  | **Mean ±SD** | **Total** | **Mean ±SD** | **Total** | **SMD (95%CI)** | ***p*** | **I^2^ (%)** | ***p*** |
| Total | 7 | 8±8 | 988 | 9±9 | 983 | -0.23 (-0.50, 0.03) | 0.082 | 85.4 | <0.001 |
| **Type of Surgery** |  |  |  |  |  |  |  |  |  |
| Liver transplantation | 1 | 8±4 | 82 | 11±5 | 80 | -0.57 (-0.88, -0.25) | <0.001 | NR | NR |
| Radical gastrectomy | 2 | 8±11 | 217 | 10±12 | 210 | -0.39 (-0.91, 0.13) | 0.142 | 86.0 | 0.008 |
| Cardiac surgery | 4 | 9±7 | 689 | 8±8 | 693 | -0.04 (-0.28, 0.20) | 0.740 | 66.6 | 0.029 |
| **Type of patient** |  |  |  |  |  |  |  |  |  |
| Adult | 6 | 9±9 | 498 | 10±11 | 494 | -0.31 (-0.56, -0.06) | 0.013 | 72.5 | 0.003 |
| Birth to 36 months | 1 | 8±7 | 490 | 7±6 | 489 | 0.15 (0.02, 0.27) | 0.020 | NR | NR |
| **Time of intervention** |  |  |  |  |  |  |  |  |  |
| Postoperative | 5 | 8±8 | 894 | 8±8 | 886 | -0.21 (-0.53, 0.11) | 0.199 | 89.3 | <0.001 |
| Intra + Post operative | 2 | 8±7 | 94 | 11±14 | 97 | -0.32 (-0.60,-0.03) | 0.030 | <0.001 | 0.489 |
| **Trigger of blood glucose(mg/dL)** | | | | | | | | | |
| ≤110 | 3 | 8±9 | 707 | 8±8 | 699 | -0.20 (-0.65, 0.25) | 0.385 | 92.1 | <0.001 |
| 110-150 | 2 | 8±7 | 94 | 11±14 | 97 | -0.32 (-0.60,-0.03) | 0.030 | <0.001 | 0.489 |
| ≥150 | 2 | 9±6 | 187 | 10±5 | 187 | -0.24 (-0.87,0.40) | 0.460 | 89.3 | 0.002 |
| **Preoperative diabetes** | | | | | | | | | |
| Yes | 4 | 8±5 | 319 | 9±5 | 316 | -0.34 (-0.71, 0.04) | 0.075 | 81.4 | 0.001 |
| No | 3 | 8±9 | 669 | 8±10 | 667 | -0.09 (-0.40,0.22) | 0.568 | 79.8 | 0.007 |
| **Use of glucocorticoids in hospital** | | | | | | | | | |
| Yes | 2 | 8±7 | 572 | 8±6 | 569 | -0.19 (-0.89, 0.51) | 0.588 | 94.2 | <0.001 |
| No | 6 | 8±10 | 416 | 10±12 | 414 | -0.26 (-0.53,0.01) | 0.061 | 72.4 | 0.006 |

Total, The number of the total patients; SMD, standardised mean difference; LOS, length of hospital stay; NA, not reported.
