# Supplementary material for: Postoperative tight glycemic control significantly reduces postoperative infection rates in patients undergoing surgery: a meta-analysis
Source: BMC Endocr Disord. 2018 Jun 22;18:42. doi: 10.1186/s12902-018-0268-9 (PMC6013895; doi:10.1186/s12902-018-0268-9)
Supplement: Supplementary file 28 — Table S17. Meta-regression for the outcome of the risk of postoperative length of hospitalization. (DOC 45 kb) [file 12902_2018_268_MOESM28_ESM.doc]

**Supplemental table 17. Meta-regression for the outcome of the risk of post-operative LOS stay.**

| **Sources** | **Coefficient (95%CI)** | **t** | ***P*** | **τ^2^** | **I^2^ Res (%)** | **Adjusted R^2^ (%)** |
| --- | --- | --- | --- | --- | --- | --- |
| Type of surgery | - 0.18 (-0.51, 0.15) | -1.40 | 0.220 | 0.068 | 77.82 | 19.65 |
| Type of patient | 0.46 (-0.27, 1.19) | 1.61 | 0.169 | 0.062 | 72.52 | 27.03 |
| Time of intervention | -0.10 (-0.89, 0.69) | -0.32 | 0.765 | 0.099 | 86.77 | -16.87 |
| Trigger of blood glucose | -0.03 (-0.43, 0.38) | -0.16 | 0.877 | 0.104 | 86.48 | -22.69 |
| Preoperative diabetes | -0.24 (-0.87, 0.39) | -0.97 | 0.376 | 0.082 | 80.81 | 3.23 |
| Use of glucocorticoids in hospital | 0.08 (-0.66, 0.83) | 0.29 | 0.786 | 0.102 | 84.21 | -20.36 |
| Jadad Score | 0.05 (-0.13, 0.23) | 0.66 | 0.537 | 0.093 | 81.02 | -9.60 |
| Year of publication | -0.02 (-0.17, 0.14) | -0.26 | 0.805 | 0.102 | 87.60 | -19.96 |
| Sample size | -0.006 (-0.0003, 0.0014) | 1.79 | 0.134 | 0.066 | 70.12 | 34.63 |
| Age | -0.006 (-0.019, 0.007) | -1.28 | 0.256 | 0.073 | 75.80 | 13.92 |

CI, Confidence interval.
